# Photoconversion of Xylan to Glyceric Acid Coupled with Hydrogen Evolution Enabled by Coral-Like S-Scheme Heterojunction

**DOI:** 10.34133/research.1399

**Published:** 2026-08-11

**Authors:** Yulong An, Yuqi Liu, Qiaoge Lu, Yiran Zhao, Fuyan Kang, Yameng Zhang, Houjuan Qi, Liquan Jing, Jinguang Hu, Zhanhua Huang

**Affiliations:** ^1^ State Key Laboratory of Woody Oil Resources Utilization, Northeast Forestry University, Harbin 150040, China.; ^2^ Key Laboratory of Bio-based Material Science and Technology, Ministry of Education, College of Material Science and Engineering, Northeast Forestry University, Harbin 150040, China.; ^3^ School of Environmental Science and Engineering, Tianjin University, Tianjin 300350, China.; ^4^ Department of Chemical and Petroleum Engineering, University of Calgary, Calgary, Alberta T2N1N4, Canada.

## Abstract

Photocatalytic biomass refining offers a promising strategy for the sustainable co-production of high-value chemicals and clean fuels. Herein, a coral-like bifunctional g-C_3_N_4_/Mn_0.7_Cd_0.3_S S-scheme heterojunction (CNMCS) was rationally constructed for simultaneous xylan photoreforming and hydrogen evolution. X-ray diffraction and transmission electron microscopy analyses confirm the formation of an interconnected hierarchical structure with intimate interfacial contact between g-C_3_N_4_ nanotubes and Mn_0.7_Cd_0.3_S nanoparticles, while x-ray photoelectron spectroscopy and density functional theory calculations reveal an S-scheme charge transfer pathway driven by the internal electric field. The optimized 6CNMCS photocatalyst achieves a glyceric acid yield of 77.6% and an H_2_ evolution rate of 2.89 mmol•g_cat_^−1^•h^−1^ without sacrificial agents. The enhanced performance is attributed to efficient charge separation and synergistic interfacial interactions that promote xylan adsorption and selective C–H bond activation. Mechanistic studies indicate that carbon-centered radicals and reactive oxygen species cooperatively drive selective C–C bond cleavage toward glyceric acid formation. This work provides a new strategy for integrating biomass valorization with clean hydrogen production.

## Introduction

Rapid industrialization and growing energy demand have intensified environmental pollution and fossil resource depletion, creating an urgent need for sustainable technologies for chemical production and energy conversion [1–3]. Biomass is the only renewable carbon resource capable of simultaneously addressing carbon neutrality and energy sustainability goals. Among various biomass feedstocks, xylan, as the major component of hemicellulose, is one of the most abundant natural polysaccharides and has attracted increasing attention as a precursor for value-added chemicals [4,5]. Efficient utilization of xylan remains challenging due to its complex polymeric structure, recalcitrant β-1,4-glycosidic bonds, and the difficulty of achieving selective bond cleavage under mild conditions [6,7].

Photocatalytic conversion has emerged as a promising strategy for biomass valorization because it directly utilizes solar energy and enables coupled oxidation–reduction reactions under mild conditions [8,9]. Compared with conventional thermochemical and biological routes, photocatalysis provides unique opportunities to regulate reaction pathways through photogenerated charge carriers and reactive intermediates while simultaneously producing value-added chemicals and clean fuels. Recent advances in photocatalytic nanomaterials have further expanded their applications in energy conversion and biomass upgrading [10–13]. In particular, photocatalytic reforming of xylan and xylose enables the production of high-value products such as furfural, xylonic acid, lactic acid, and glyceric acid (GA) [14,15]. Among these products, GA has attracted increasing attention as an important platform molecule owing to its high added value and broad industrial relevance [16,17]. However, achieving efficient and selective photocatalytic biomass conversion remains challenging. During photocatalysis, photogenerated holes and reactive oxygen species (ROS) participate in substrate activation and oxidation, whereas photogenerated electrons drive reduction reactions [18–20]. The competition between charge recombination and radical-mediated pathways often leads to reduced selectivity and carbon loss. Therefore, photocatalytic systems capable of simultaneously promoting charge separation, preserving strong redox ability, and directing selective C–C bond cleavage are highly desirable [18,21].

Bimetallic sulfides have attracted substantial attention in photocatalysis due to their tunable electronic structures, broad visible-light absorption, and abundant surface catalytic sites [22–25]. Mn*_x_*Cd_1-*x*_S solid solutions exhibit excellent intrinsic activity, where electronic properties and energy band positions can be systematically tailored through composition engineering [26–28]. In particular, Mn_0.7_Cd_0.3_S demonstrates high efficiency for visible-light-driven hydrogen evolution [15]. However, its capability to drive coupled oxidative biomass transformations is restricted by rapid carrier recombination and limited oxidative potential. Heterojunction engineering, especially the construction of S-scheme systems, provides an effective approach to overcome these limitations [21,29–34]. Unlike conventional type II heterojunctions, where photogenerated carriers migrate to lower-potential bands and lose redox power, S-scheme systems preserve strong redox capabilities through interface-driven charge transfer [35–41]. Upon intimate contact, the difference in work functions between the 2 semiconductors drives electron diffusion to align their Fermi levels [42,43]. This charge redistribution creates a built-in electric field (IEF), interfacial band bending, and an electric dipole [31,44–48]. Under illumination, the combined effect of the IEF and band bending directs the selective recombination of weaker electrons and holes. Meanwhile, highly reductive conduction-band electrons and highly oxidative valence-band holes are spatially retained [49,50]. Constructing such S-scheme architectures with bimetallic sulfides accelerates interfacial charge transfer kinetics. More importantly, it maintains the driving force required for simultaneous proton reduction and selective C–H/C–C bond cleavage in biomass feedstocks.

Graphitic carbon nitride (g-C_3_N_4_), a representative metal-free semiconductor, offers excellent chemical stability, suitable band alignment, and an intrinsic π-conjugated electronic structure [51,52]. Morphological engineering of g-C_3_N_4_ further enhances photocatalytic performance [53–56]. Hollow, porous, and hierarchical architectures improve light harvesting, shorten carrier diffusion paths, expose more active sites, and facilitate intimate heterointerface formation. Hollow nanotubular structures induce multiple internal reflections and improve mass transport, while surface amino and hydroxyl groups promote substrate adsorption and activation [52,57]. These properties make nanostructured g-C_3_N_4_ an ideal oxidation photocatalyst for constructing efficient S-scheme heterojunction systems for selective biomass photoreforming [58–65].

Inspired by natural coral architectures, we constructed a coral-like bifunctional g-C_3_N_4_/Mn_0.7_Cd_0.3_S (CNMCS) S-scheme heterojunction photocatalyst for simultaneous xylan photoreforming and hydrogen evolution without sacrificial agents. The optimized 6CNMCS converts xylan into GA with a yield of 77.6% and achieves a hydrogen evolution rate of 2.89 mmol•g_cat_^−1^•h^−1^. The enhanced performance arises from the synergistic effects of the coral-like hierarchical structure, efficient S-scheme charge transfer, and strengthened substrate–catalyst interactions, which promote selective C–H activation and directional charge utilization. Density functional theory (DFT) calculations combined with in situ characterizations reveal the interfacial charge migration behavior and the reaction pathway responsible for selective biomass conversion. Table S1 summarizes the hydrogen evolution performance compared with recently reported photocatalysts, highlighting the superior activity of CNMCS.

## Results and Discussion

**Fig. 1.**
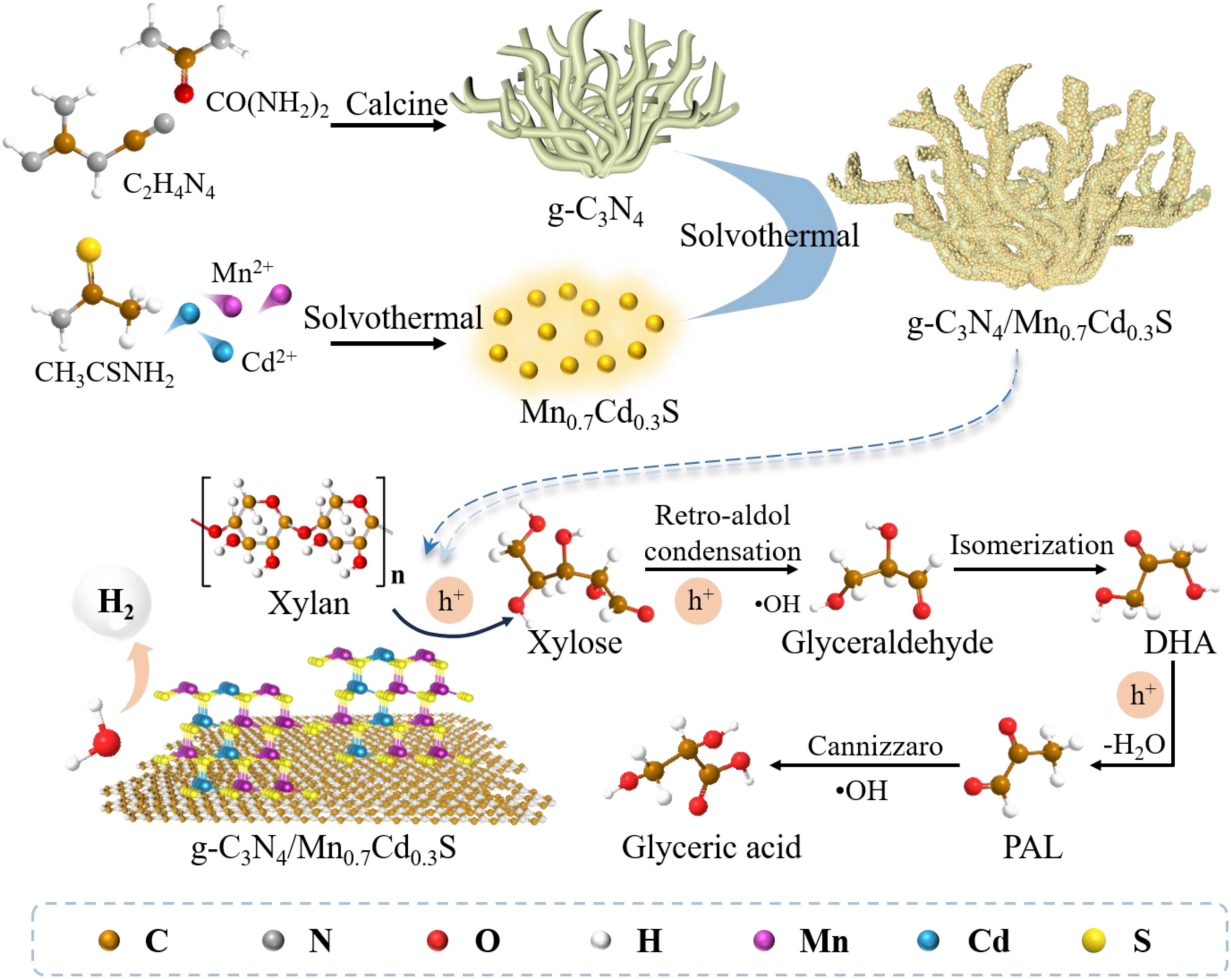
Schematic illustration depicting the preparation process and subsequent photocatalytic mechanism of the CNMCS heterojunction.

**Fig. 2.**
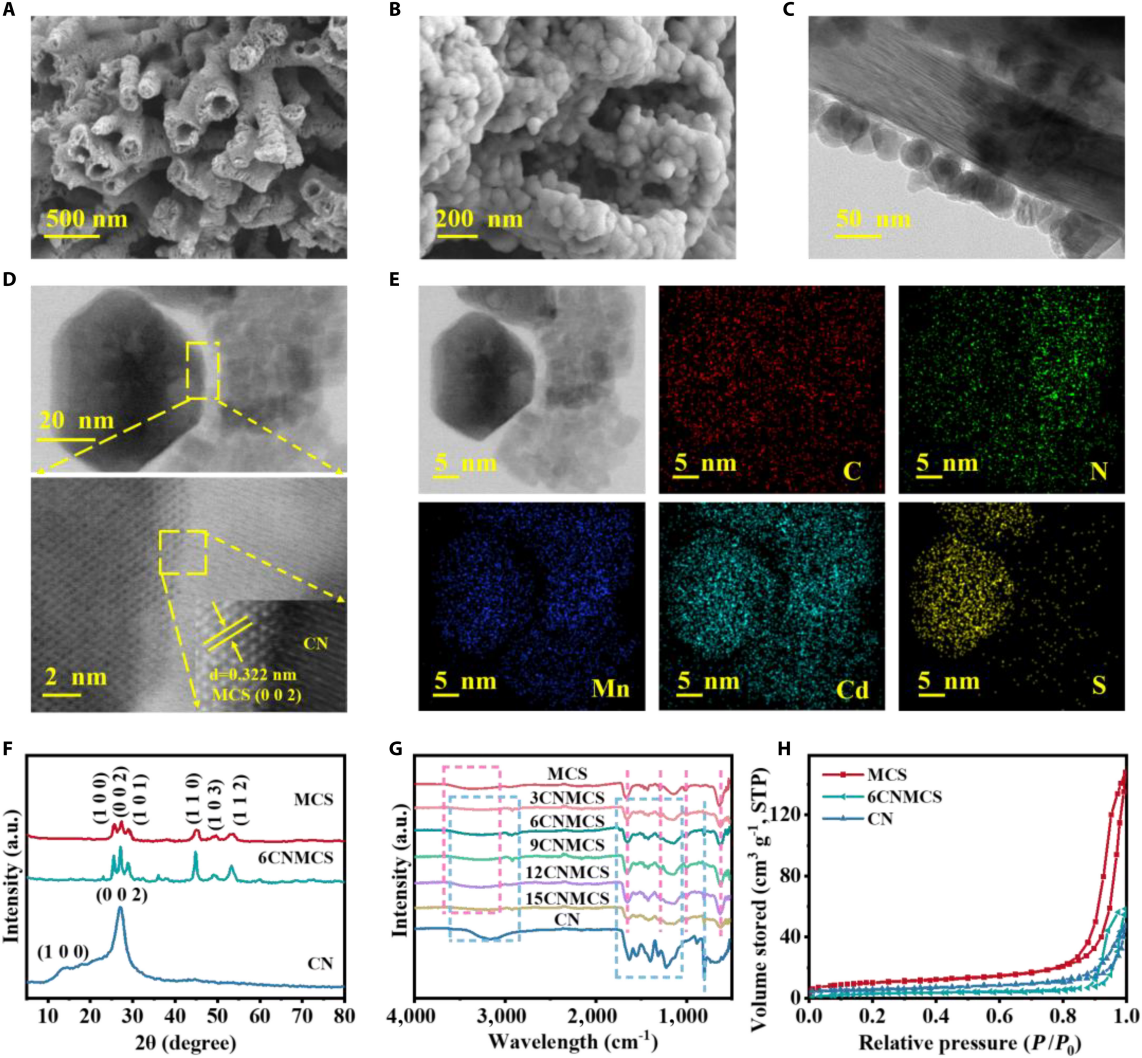
SEM images of (A) CN and (B) 6CNMCS. (C) TEM and (D) AC-HAADF-STEM images of 6CNMCS. (E) Element mapping of the prepared 6CNMCS. (F) XRD pattern, (G) FTIR spectra, and (H) N_2_ adsorption–desorption isotherms of the CN, 6CNMCS, and MCS.

A coral-like CNMCS heterostructure was successfully constructed via a simple solvothermal method (Fig. 1). Scanning electron microscopy (SEM) revealed that the as-synthesized MCS consisted of nanoparticles with diameters ranging from 20 to 50 nm (Fig. S1A and B), while CN exhibited a multi-hollow nanotube morphology (Fig. 2A and Fig. S1C). High-resolution transmission electron microscopy (HRTEM) images showed that MCS nanoparticles were uniformly anchored onto the surface of CN nanotubes, forming a coral-like structure (Fig. 2B and C). This unique architecture endows the material with a larger specific surface area and abundant active sites while simultaneously promoting internal light scattering and electron transfer, thereby synergistically enhancing the photocatalytic efficiency. Electron paramagnetic resonance (EPR) characterization indicated that CNMCS possessed more nitrogen vacancies than pristine CN (Fig. S2). This increase is primarily attributed to interfacial strain, metal-coordination etching, and changes in the local chemical environment during the formation of the composite. Aberration-corrected high-angle annular dark-field scanning transmission electron microscopy (AC-HAADF-STEM) further revealed 2 distinct lattice structures: lattice fringes with a spacing of 0.32 nm corresponding to the (002) plane of MCS (Fig. 2D). Energy-dispersive x-ray spectroscopy (EDS) elemental mapping (Fig. 2E) showed that C, N, Mn, Cd, and S were uniformly distributed throughout the entire structure, confirming the successful formation of the composite.

X-ray diffraction (XRD) patterns (Fig. 2F) exhibited diffraction peaks at 2θ = 25.5°, 27.3°, 28.9°, 45.1°, 49.6°, and 52.3°, corresponding to the (100), (002), (101), (110), (103), and (112) crystal planes of MCS (PDF#00-040-1289) [28]. The characteristic peaks of CN corresponding to the (100) and (002) planes were observed at 13.3° and 27.0°, respectively (PDF#01-087-1526) [28]. In the XRD pattern of 6CNMCS, only diffraction peaks of MCS were detected, which is attributed to the low CN content and the overlap between the CN (002) peak (27.0°) and the MCS (002) peak (27.3°), while also confirming the high phase purity of the composite. Fourier transform infrared (FTIR) spectroscopy was employed to identify key chemical functional groups (Fig. 2G). Pristine CN exhibited a stretching vibration of the tri-s-triazine ring at 806 cm^−1^, while the absorptions in the range of 1,015 to 1,751 cm^−1^ corresponded to the stretching modes of C–N bonds in the aromatic heterocycle. A broad absorption band from 2,840 to 3,600 cm^−1^ originated from O–H and N–H stretching vibrations [66]. For MCS, the absorptions at 1,653 cm^−1^ and in the range of 3,055 to 3,682 cm^−1^ were attributed to the O–H stretching vibration of surface-adsorbed water; the characteristic vibration at 636 cm^−1^ corresponded to the Mn–S bond, and the absorptions at 1,007 and 1,289 cm^−1^ were assigned to the vibrational modes of the Cd–S bond [67]. The FTIR spectrum of CNMCS contained the characteristic peaks of both CN and MCS, confirming the successful construction of the composite.

**Fig. 3.**
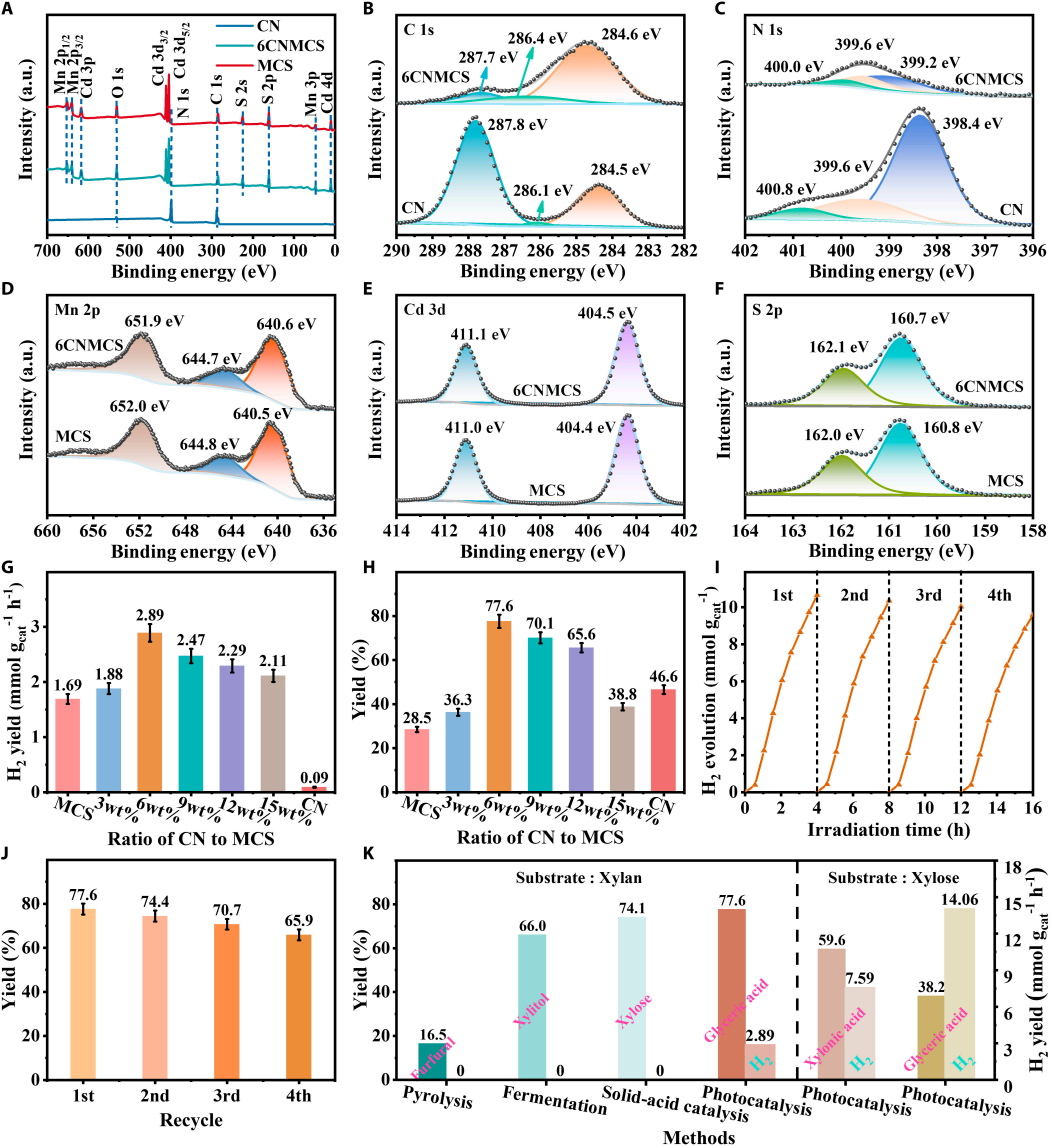
(A) XPS survey spectrum, (B) C 1s, (C) N 1s, (D) Mn 2p, (E) Cd 3d, and (F) S 2p high-resolution spectra of the 6CNMCS, CN, and MCS. Effect of different catalysts on (G) the H_2_ evolution rate during photocatalytic water splitting and (H) the yield of glycolic acid from the photocatalytic conversion of xylan. (I and J) Photocatalytic cycle performance testing of 6CNMCS. (K) Comparison of gas-phase and liquid-phase products from the reforming of xylose and xylan using pyrolysis, fermentation, catalysis, and photocatalysis methods.

To elucidate the surface chemical states and electronic interactions, x-ray photoelectron spectroscopy (XPS) analysis was performed on the 6CNMCS composite (Fig. 3A to F). The survey spectrum (Fig. 3A) confirms the presence of C, N, Mn, Cd, and S elements, which is in good agreement with the EDS results, verifying the successful construction of the composite. High-resolution spectra provide further insight into the chemical environments of the constituent elements. The C 1s spectrum (Fig. 3B) can be deconvoluted into 3 peaks located at 287.8, 286.1, and 284.5 eV, corresponding to N–C=N, C–NH_2_, and C–C bonds, respectively [30,38]. Similarly, the N 1s spectrum (Fig. 3C) is fitted into 3 components at 400.8 eV (-N–H), 399.6 eV [N-(C)_3_], and 398.4 eV (C=N–C) [28,66]. Notably, compared with pristine CN, an additional component appears at 286.5 eV in the C 1s spectrum of 6CNMCS, which may originate from changes in the local electronic environment induced by heterojunction formation. Meanwhile, slight shifts in the binding energies of both C 1s and N 1s are observed, indicating electron-density redistribution and electronic interactions at the CN/MCS interface. For the MCS component, the Mn 2p spectrum (Fig. 3D) exhibits 2 characteristic peaks at 640.5 eV (Mn 2p_3/2_) and 652.0 eV (Mn 2p_1/2_), along with a satellite peak at 644.8 eV, confirming the presence of Mn^2+^ species [68,69]. The Cd 3d spectrum (Fig. 3E) shows 2 distinct peaks at 404.4 eV (Cd 3d_5/2_) and 411.0 eV (Cd 3d_3/2_), while the S 2p spectrum (Fig. 3F) can be resolved into S 2p_3/2_ and S 2p_1/2_ peaks located at 160.8 and 162.0 eV, respectively [67,70]. In 6CNMCS, additional components are observed in the Cd 3d spectrum at 404.8 and 411.4 eV, which may be associated with interfacial coordination effects or charge redistribution arising from heterojunction formation. Concurrently, subtle shifts are observed in the binding energies of the Mn 2p and S 2p peaks. Collectively, these XPS results confirm the successful construction of the CNMCS heterostructure and suggest the presence of substantial electronic interactions and possible interfacial coordination between CN and MCS, which are beneficial for interfacial charge transfer.

The textural properties of CN, MCS, and 6CNMCS were investigated by N_2_ adsorption–desorption measurements (Fig. 2H, Fig. S3, and Table S2). All samples exhibit typical type IV isotherms with H3 hysteresis loops, confirming the presence of mesoporous structures [27]. This mesoporous characteristic is consistent with the coral-like hierarchical morphology observed in the SEM and TEM images. Notably, the porous structure mainly originates from the hollow nanotubular architecture of CN, nanotube stacking, and interparticle voids generated between CN nanotubes and MCS nanoparticles, rather than from highly ordered intrinsic mesoporous frameworks. The pore-size distribution curves reveal distinct structural characteristics for each sample. MCS exhibits a dominant pore distribution in the range of 23 to 52 nm, while CN shows a broader distribution from 6 to 60 nm due to its hollow nanotubular structure and intertube voids. In comparison, 6CNMCS presents a more concentrated mesoporous distribution centered at 17 to 39 nm, indicating the formation of a more uniform porous network after heterojunction construction. Such mesoporous channels facilitate reactant diffusion and mass transfer during photocatalytic reactions. The specific surface areas of MCS, 6CNMCS, and CN are 23.17, 19.75, and 13.06 m^2^•g^−1^, respectively. Although these values are moderate, they are consistent with the observed morphology. More importantly, the markedly enhanced photocatalytic activity of 6CNMCS cannot be attributed solely to surface-area effects but is mainly associated with the efficient charge separation and interfacial charge transfer behavior enabled by the S-scheme heterojunction.

It should be noted that no sacrificial agent was introduced in this work. Instead, xylan was intentionally employed as both the biomass substrate and hole acceptor, allowing photogenerated holes to directly participate in selective xylan oxidation rather than being consumed by sacrificial reagents. This design enables the simultaneous co-production of hydrogen and value-added GA and represents an important feature of the CNMCS photocatalytic system. The photocatalytic hydrogen evolution and xylan oxidation performances of CN, CNMCS, and MCS were evaluated under visible light using xylan as the hole scavenger (Fig. S4). As the CN content increases, the hydrogen evolution activity of CNMCS first improves and then declines, reaching an optimum at 6 wt % CN (Fig. 3G and H). The optimal hydrogen evolution rate of 6CNMCS is 2.89 mmol•g_cat_^−1^•h^−1^, which is 1.7 and 32.1 times higher than those of pure MCS and CN, respectively. Meanwhile, the introduction of CN also plays a critical role in GA production. The 6CNMCS composite achieves the highest GA yield of 77.6%, which is 2.72 and 1.67 times greater than those of MCS and CN. However, excessive CN loading leads to a decline in product yield, indicating the importance of optimizing the component ratio. Reaction conditions were further optimized as shown in Figs. S5 to S7. The optimal amounts of NaOH, xylan, and photocatalyst were determined to be 5.0 g, 100 mg, and 25 mg, respectively. In addition, CNMCS exhibits excellent catalytic performance in the photorefining of various biomass-derived substrates, including xylose, arabinose, glucose, fructose, and glucan (Fig. S7). The recyclability of the catalyst was also evaluated. After 4 consecutive cycles, 6CNMCS retains 85% of its initial GA yield and 91% of its hydrogen evolution capacity (Fig. 3I and J), demonstrating its high stability and resistance to photocorrosion, as confirmed by XRD (Fig. S9) and XPS (Fig. S10) analyses showing preserved crystal structure and surface chemical states, and by inductively coupled plasma mass spectrometry (ICP-MS) measurement indicating minimal Cd^2+^ leaching (0.81 mg•l^−1^, <0.5% of total Cd). Compared with conventional thermochemical and biological processes, this photocatalytic system offers the dual advantage of high-value product generation and simultaneous hydrogen production [24,25]. As summarized in Fig. 3K and Table S3, the CNMCS system exhibits superior selectivity, yield, and hydrogen evolution rate for the photoreforming of structurally complex xylan, even when compared with systems using simpler substrates such as xylose. This outstanding performance can be attributed to the intrinsically enhanced photocatalytic properties of the CNMCS heterostructure. Here, we concentrate on the formation of GA and hydrogen; quantification of other carbon-containing products is not included.

**Fig. 4.**
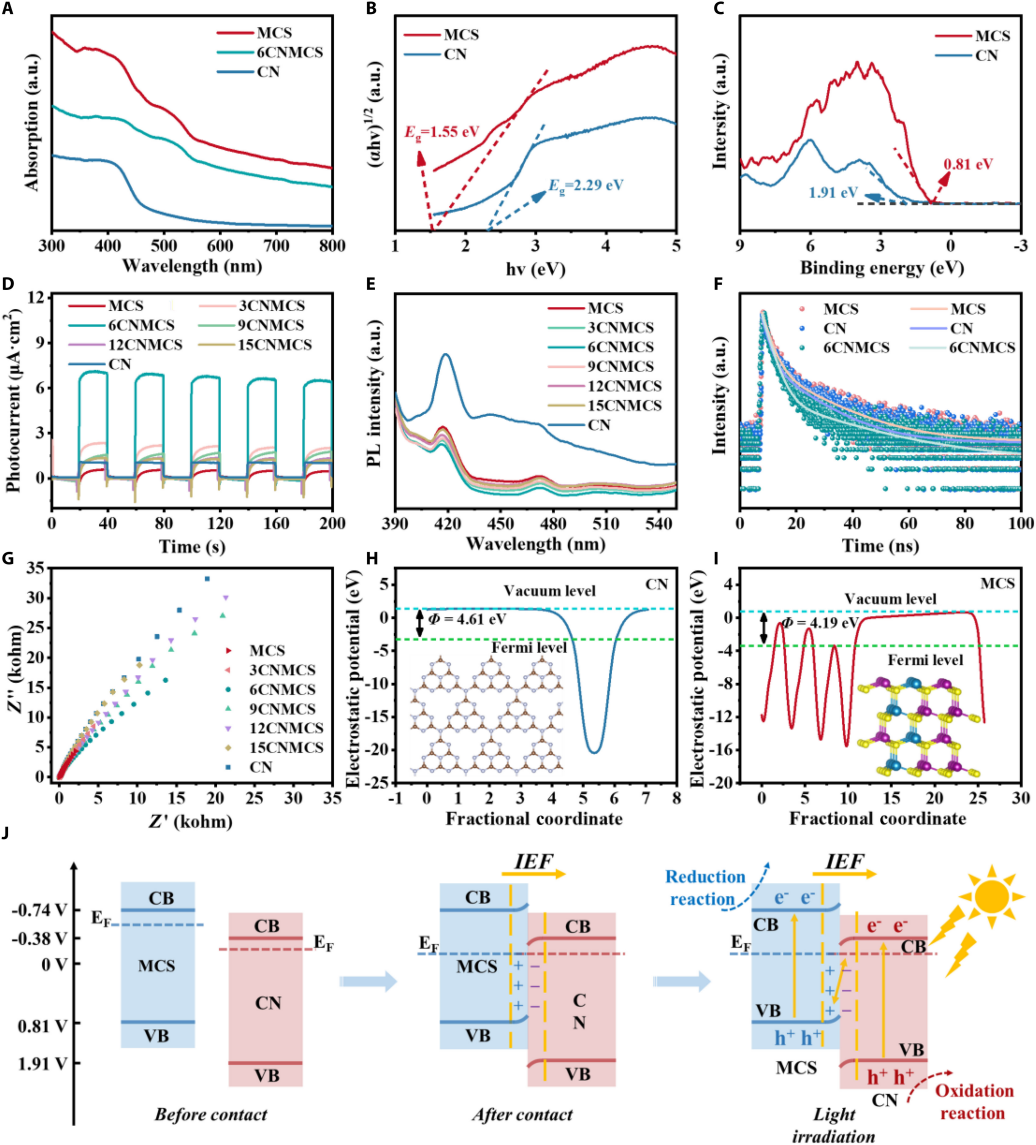
(A) UV–Vis diffuse reflectance spectroscopy and (B) Tauc plots of CN, CNMCS, and MCS. (C) VB-XPS spectra of CN and MCS. (D) Transient photocurrent response, (E) PL spectrum, and (F) TRPL decay curves of the prepared CN, CNMCS, and MCS. (G) EIS plots of CN, CNMCS, and MCS. The calculated work functions of (H) CN and (I) MCS. (J) Possible flow paths of electrons.

The optical properties and electronic band structures of the samples were investigated by ultraviolet–visible (UV–Vis) diffuse reflectance spectroscopy as shown in Fig. 4A and Fig. S11. The absorption edges of MCS and CN are located at approximately 589 and 481 nm, respectively. Compared with the pristine components, CNMCS exhibits an obvious red shift in the absorption edge, with 6CNMCS showing the most pronounced shift, indicating enhanced visible-light absorption and suppressed charge recombination [28]. The band gap energies were estimated using the Kubelka–Munk method based on Tauc plots. The band gaps of CN and MCS are determined to be 2.29 and 1.55 eV, respectively as shown in Fig. 4B. To further determine the band structure, valence band XPS (VB-XPS) measurements were carried out. The VB maxima of CN and MCS are located at 1.91 and 0.81 V [versus normal hydrogen electrode (NHE)], respectively, as shown in Fig. 4C. Accordingly, the conduction band (CB) positions are calculated to be −0.38 V for CN and −0.74 V for MCS (versus NHE) [22]. These results provide a solid basis for understanding the band alignment and confirm the successful construction of the CN/MCS heterostructure.

Transient photocurrent measurements (Fig. 4D) reveal that MCS exhibits a substantially lower photocurrent density than CN, indicative of severe charge carrier recombination. In contrast, the CNMCS composites deliver a markedly enhanced photocurrent response, suggesting that the introduction of the heterointerface promotes more efficient charge carrier extraction and migration under illumination [70]. To gain further insight into the recombination dynamics, photoluminescence (PL) spectra were analyzed over an extended wavelength range (Fig. 4E). Pristine CN exhibits the strongest emission intensity with a dominant emission band centered at approximately 418 nm, reflecting pronounced radiative recombination, whereas MCS displays a relatively weaker signal. A weaker secondary emission feature around 471 nm is also observed for all samples, which may be associated with surface or interfacial electronic states. Notably, the overall PL intensity is progressively quenched in the CNMCS composites across the entire measured spectral range, with 6CNMCS exhibiting the lowest emission intensity. This pronounced quenching indicates that the formation of the heterointerface effectively suppresses radiative electron-hole recombination and facilitates charge separation through interfacial electronic interactions [71]. Time-resolved photoluminescence (TRPL) measurements (Fig. 4F and Table S4) provide additional evidence for the altered carrier dynamics. The average decay lifetime of CNMCS is shortened to 1.49 ns compared with CN and MCS, implying that photoexcited carriers are rapidly funneled into nonradiative pathways associated with interfacial charge transfer processes rather than undergoing recombination. Furthermore, electrochemical impedance spectroscopy (EIS) Nyquist plots (Fig. 4G) show that 6CNMCS possesses the smallest semicircle radius among all samples, corresponding to the lowest charge transfer resistance [72]. This result highlights the accelerated interfacial electron transport kinetics in the heterostructure. Collectively, these results consistently demonstrate that the construction of the CNMCS heterojunction effectively modulates charge carrier dynamics by suppressing radiative recombination, accelerating interfacial charge transfer, and improving charge transport efficiency, thereby contributing to the enhanced photocatalytic performance.

The charge separation pathway in the CNMCS heterojunction was investigated to confirm its S-scheme character [73]. DFT calculations provided initial insight into the electronic structure, revealing a higher work function (*Φ*) for CN (4.61 eV) than for MCS (4.19 eV) (Fig. 4H and I). This difference establishes the thermodynamic driving force for electron transfer from MCS to CN upon intimate contact, leading to Fermi-level equilibration at the interface [74]. As a result, downward band bending occurs in CN, while upward band bending is formed in MCS, generating an internal electric field directed from MCS to CN. To experimentally verify this theoretical prediction, ultraviolet photoelectron spectroscopy (UPS) measurements were conducted. The UPS spectra (Fig. S12) show secondary electron cutoff energies corresponding to work functions of 4.54 eV for CN and 4.10 eV for MCS, closely matching the DFT-calculated values. These results provide direct experimental evidence that electron transfer occurs from MCS to CN, corroborating the proposed internal electric field direction and S-scheme charge transfer pathway. Under visible-light irradiation, the IEF, together with band bending and Coulombic interactions, drives the selective recombination of photogenerated electrons in the CB of CN with holes in the VB of MCS at the heterointerface, which is characteristic of an S-scheme charge transfer pathway. Consequently, strongly reductive electrons are preserved in the CB of MCS, while strongly oxidative holes remain in the VB of CN, enabling efficient reduction and oxidation reactions for photocatalytic hydrogen evolution and selective xylan photoreforming.

This proposed mechanism is further supported by in situ XPS analysis (Fig. S13), which probes changes in surface electron density under illumination. Positive binding-energy shifts are observed for CN (C 1s and N 1s), whereas negative shifts are detected for MCS (Mn 2p, Cd 3d, and S 2p). These opposite binding-energy shifts provide strong evidence for the light-induced interfacial electron transfer behavior consistent with the proposed S-scheme mechanism. Compared with conventional type II and traditional Z-scheme heterojunctions, the CNMCS system exhibits charge transfer behavior and redox characteristics that are more consistent with the S-scheme mechanism, as summarized in Table S5. Based on the above findings, the charge separation and migration mechanism of the CNMCS heterojunction is illustrated in Fig. 4J.

**Fig. 5.**
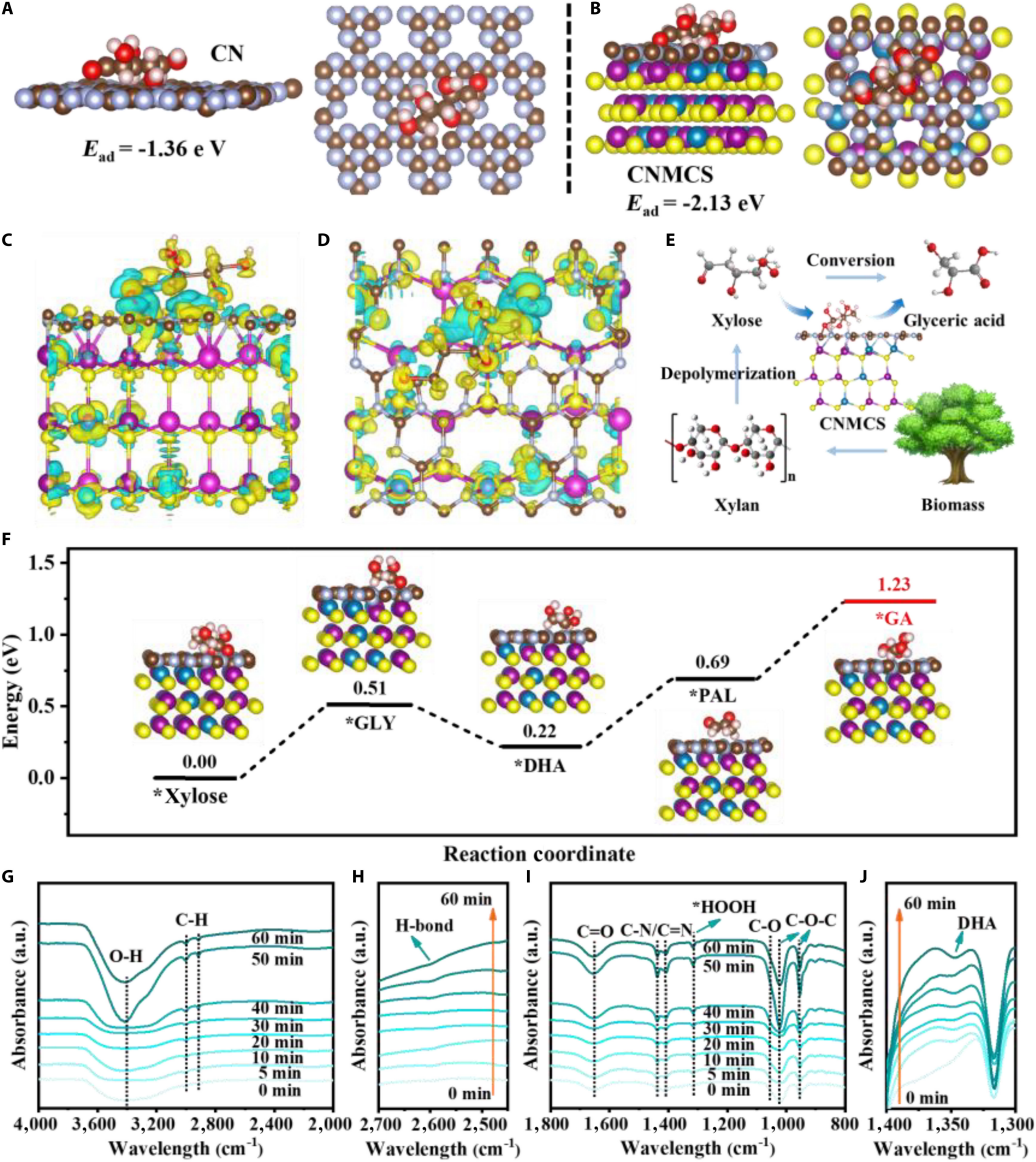
Calculated adsorption of xylose on (A) CN and (B) CNMCS. (C and D) Charge density difference maps of the as-obtained samples. The yellow and cyan regions represented the electron accumulation and depletion, respectively. The values at the atoms were the Bader charge. (E) Schematic illustration of photocatalysis. (F) Calculated reaction energy diagram for the reaction paths followed by the xylose conversion into GA on CNMCS. (G to J) In situ FT-IR spectra of CNMCS for the adsorption of xylan in the dark and under light illumination for another 1 h.

In an alkaline reaction medium, the synergistic effect of photogenerated •OH radicals and nucleophilic attack by OH^−^ promotes the cleavage of β-1,4 glycosidic bonds in xylan, yielding xylose, xylobiose, and other xylo-oligosaccharide fragments [75–77]. Control experiments (Fig. S14) showed that xylan underwent only limited depolymerization in NaOH solution in the absence of light, whereas the degree of depolymerization increased substantially upon illumination, confirming the critical role of photogenerated reactive species in glycosidic bond cleavage. Consequently, the photocatalytic reforming of xylan follows a 2-step depolymerization–conversion mechanism. In the first step, photogenerated •OH radicals drive the depolymerization of xylan into reducing sugar units under alkaline conditions. Subsequently, these sugar units undergo selective C–C bond cleavage mediated by photogenerated holes and ROS on the CNMCS surface, leading to the formation of GA. Based on this understanding, xylose was selected as a model substrate for DFT calculations, as it represents the core structural unit of xylan depolymerization products, and its C–C bond cleavage mechanism is a key step in the photocatalytic pathway. Reasonable thin-layer models of CN and CNMCS heterojunctions were constructed (Figs. S15 and S16). The adsorption energies of xylose on CN and CNMCS surfaces were determined to be −1.36 and −2.13 eV, respectively (Fig. 5A and B and Fig. S17). The considerably higher adsorption energy on the heterojunction surface indicates stronger interaction with the substrate, which is more favorable for the activation of C–H bonds. To gain deeper insight into the activation mechanism of xylose on the CNMCS heterojunction, charge density difference simulations were performed (Fig. 5C and D). Owing to the intimate interfacial contact formed between CN and MCS, a redistribution of electrons occurs at the interface. Specifically, the surface of MCS is dominated by yellow regions indicative of electron accumulation, whereas the CN surface primarily exhibits blue regions corresponding to electron depletion. This polarized charge distribution renders the electron-deficient regions on the CN surface as preferential accumulation sites for photogenerated holes, generating localized positively charged domains at the interface. These regions can electrostatically attract the electron cloud surrounding the β-H site of xylose, thereby facilitating C–H bond activation [78]. Local charge transfer analysis of the adsorption configuration further reveals that electrons are primarily concentrated in the lone pair regions of oxygen atoms in xylose, while electron depletion is observed on the π-conjugated system of CN. This directly confirms the existence of n-π* interactions, which serve as the key driving force for stable xylose adsorption on the catalyst surface. Compared with pristine CN, the CNMCS heterojunction on one hand activates the β-H site through interface-induced hole enrichment, and on the other hand enhances the interfacial anchoring capability for xylose via n-π* interactions. This synergistic effect markedly improves the adsorption affinity toward xylose, promotes the selective cleavage of the C–C bond adjacent to the β-H site, and consequently boosts the photocatalytic conversion efficiency.

DFT calculations were further employed to investigate the photocatalytic conversion process of xylose to GA over the CNMCS heterojunction. The proposed reaction pathway involves multiple elementary steps (Fig. 5F and Fig. S18):

1. Adsorption of xylose onto the CNMCS surface (C_5_H_10_O_5_*, 0 eV).
2. Conversion of xylose via retro-aldol condensation to generate glyceraldehyde (GLY) (C_3_H_6_O_3_*, 0.51 eV).
3. Isomerization of GLY to 1,3-dihydroxyacetone (DHA) (C_3_H_6_O_3_*, 0.22 eV).
4. Dehydration of DHA to form pyruvaldehyde (PAL) (C_3_H_4_O_3_*, 0.69 eV).
4. A cross-Cannizzaro reaction between PAL and GLY, which contains only one α-H atom, yielding GA (C_3_H_6_O_4_*, 1.23 eV).

This pathway illustrates the energetics of the stepwise transformation from xylose to GA on the CNMCS heterojunction.

In situ FTIR spectroscopy was employed to monitor the evolution of functional groups during the reaction (Fig. 5G to J and Fig. S19). The intensity of the C–H vibrational peaks at 2,920 and 3,000 cm^−1^ increased slightly over time, which is attributed to the gradual depolymerization of the xylan backbone and the accumulation of intermediates containing C–H structures [79]. These C–H vibrational peaks exhibited a slight redshift, indicating a weakening of the C–H bonds (Fig. S19). This phenomenon is likely due to C–H/π interactions between xylan or xylose and the π-conjugated system of the catalyst, which facilitate the anchoring of substrates on the catalyst surface and the pre-activation of the targeted C–H bonds. More importantly, the C–H/π interactions induce polarization of the target C–H bonds, leading to a redistribution of intramolecular electron density. This subsequently weakens the C–C bonds connected to the relevant carbon atoms, thereby establishing a critical foundation for the subsequent selective cleavage of C–C bonds. The O–H vibrational peak at 3,408 cm^−1^ underwent noticeable changes with reaction time, reflecting the dynamic reconstruction of the interfacial hydrogen bonding network [79]. During the initial stage of the photocatalytic reaction (0 to 40 min), xylose molecules formed interfacial hydrogen bonds with the amino or hydroxyl functional groups on the CNMCS surface through their -OH groups. The coexistence and gradual homogenization of multiple hydrogen bonding states resulted in a progressive broadening and flattening of the O–H peak, indicating the establishment and saturation of the hydrogen bonding network. As the reaction proceeded, a substantial amount of -OH-containing intermediates such as GLY and DHA were generated. Their relatively consistent O–H coupled vibrational characteristics overlapped with the original broad peak, causing the peak shape to abruptly sharpen. With further reaction, the newly formed -OH-containing intermediates continued to participate in the reconstruction of the interfacial hydrogen bonding network. However, the complexity and uniformity of the network at this stage were inferior to those in the initial phase, leading to only a slight recovery in peak shape that could hardly revert to the original flattened top state. The peak at 2,598 cm^−1^ is assigned to O–H stretching vibrations involved in strong hydrogen bonding, corresponding to the dimers formed from carboxylic acid products (Fig. 5H) [79]. Its emergence and intensification confirm the stable accumulation of GA in the reaction system. The broad peak in the region of 1,600 to 1,700 cm^−1^ corresponds to C=O stretching vibrations, encompassing contributions from carbonyl-containing intermediates and the product GA. The absorption peak at 1,200 cm^−1^ is attributed to *HOOH species, indicating the photogeneration of abundant ROS such as •OOH [79]. The absorptions in the range of 950 to 1,100 cm^−1^ arise from C–O and C–O–C stretching vibrations. The gradual increase in absorption intensity in this region over the course of the reaction is ascribed to the exposure of C–O–C bonds during xylan depolymerization and the formation of oxygenated products. The characteristic peak at 1,335 cm^−1^ is associated with the photocatalytic conversion products of DHA [79]. Its gradually increasing intensity indicates that DHA, as a key intermediate, is further oxidized to GA.

**Fig. 6.**
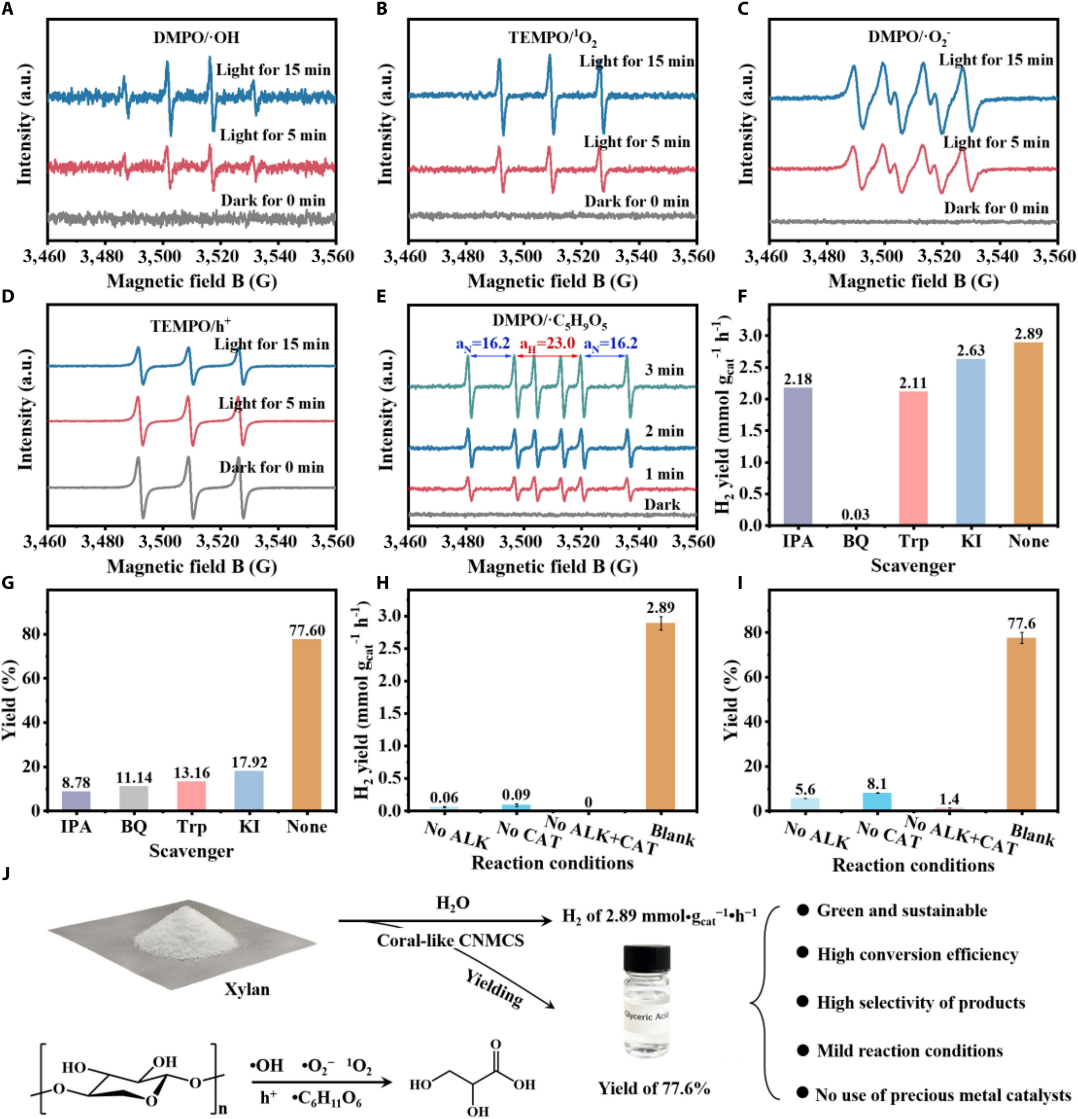
The corresponding ESR spectra for the detection of (A) DMPO/•OH, (B) DMPO/•O_2_^−^, (C) TEMP/^1^O_2_, (D) TEMPO/h^+^, and (E) TEMPO/•C_6_H_11_O_6_ adducts are shown. Photocatalytic reforming of xylan over 6CNMCS. (F) H_2_ production and (G) GA yield measured under radical trapping conditions. Control experiments for (H) H_2_ production and (I) GA yield was conducted in the absence of alkali (No ALK), catalyst (No CAT), or both (No ALK + CAT). (J) Schematic illustration of the efficient and sustainable co-production of hydrogen and GA by coral-like CNMCS.

To identify the key reactive species involved in the process of photocatalytic xylan conversion coupled with hydrogen production via water splitting, radical trapping experiments were conducted under optimal conditions. Isopropanol (IPA), benzoquinone (BQ), l-tryptophan (Trp), and potassium iodide (KI) were employed as scavengers for •OH, •O_2_^−^, ^1^O_2_, and h^+^, respectively [25]. As shown in Fig. 6F, hydrogen evolution was almost completely suppressed after adding BQ, indicating that •O_2_^−^ plays a dominant role in the hydrogen evolution process, whereas •OH, ^1^O_2_, and h^+^ contribute to a lesser extent. For GA production (Fig. 6G), all scavengers caused substantial decreases in yield, suggesting the synergistic involvement of multiple reactive species. Among them, IPA and BQ exhibited stronger inhibition effects, indicating that •OH and •O_2_^−^ are the primary active species responsible for xylan oxidation and selective C–C bond cleavage, while ^1^O_2_ and h^+^ participate in substrate activation and intermediate transformation. Electron spin resonance (ESR) spectroscopy was further employed to verify the generation of these reactive species [80]. 5,5-Dimethyl-1-pyrroline N-oxide (DMPO), 2,2,6,6-tetramethylpiperidine (TEMP), and 2,2,6,6-tetramethylpiperidinyloxy (TEMPO) were used as spin traps to detect •OH, •O_2_^−^, ^1^O_2_, and h^+^, respectively. No signals were detected under dark conditions, confirming that all processes were light-driven. Upon visible-light irradiation, characteristic signals were immediately detected. These included the 1:2:2:1 quartet of DMPO/•OH (Fig. 6A), the sextet of DMPO/•O_2_^−^ (Fig. 6B), and the 1:1:1 triplet of TEMP/^1^O_2_ (Fig. 6C). Under continuous illumination, the intensities of all signals increased over time, directly demonstrating the accelerated generation of •OH, •O_2_^−^, and ^1^O_2_ during the photocatalytic reaction [23,81]. For the detection of h^+^, the 1:1:1 triplet signal of TEMPO/h^+^ diminished in intensity after illumination (Fig. 6D), which is attributed to the consumption of TEMPO by photogenerated electrons to form 1-hydroxy-2,2,6,6-tetramethylpiperidine (TEMPOH) [25]. To further investigate the key intermediates in the xylan conversion process, DMPO was used to trap carbon-centered radicals. As shown in Fig. 6E, a characteristic sextet ESR signal was detected after 1 min of illumination, with hyperfine splitting parameters of a_N_ = 16.2 G and a_H_ = 23.0 G. These parameters are in good agreement with those reported in the literature for the DMPO adduct of carbon-centered radicals [82]. The signal intensity increased with prolonged irradiation time, indicating the continuous accumulation of carbon-centered radicals. This result confirms that photogenerated holes abstract hydrogen atoms from pre-activated C–H bonds, generating •C_5_H_9_O_5_ carbon-centered radical intermediates. Collectively, these results demonstrate that •OH, •O_2_^−^, ^1^O_2_, h^+^, and e^−^ are the primary reactive species in this photocatalytic system, with carbon-centered radicals serving as key reaction intermediates. Because the reaction vessel has been vacuumed before illumination, the active species are probably produced in this way:

$$hv\to h^{+}+e^{-}$$ (1)

$$H_{2}O+h^{+}\to •\mathrm{OH}+O_{2}$$ (2)

$${\mathrm{OH}}^{-}+h^{+}\to •\mathrm{OH}$$ (3)

$$•\mathrm{OH}/O_{2}+e^{-}\to •{O_{2}}^{-}$$ (4)

$$•{O_{2}}^{-}+h^{+}\to {}^{1}O_{2}$$ (5)

The alkaline medium plays a dual role in this reaction system. A high concentration of NaOH can modify the surface potential of S-based materials, thereby facilitating the water oxidation pathway (3) [83,84]. The in situ generated O_2_ is reduced by photogenerated electrons to form ROS such as •O_2_^−^ and ^1^O_2_, which further participate in the oxidative conversion of xylan. Additionally, OH^−^ exhibits nucleophilic attack capability toward β-1,4 glycosidic bonds, assisting in bond cleavage and enabling the depolymerization of xylan into xylose units [85,86]. To elucidate the respective contributions of the alkali and the photocatalyst, control experiments were conducted (Fig. 6H and I). The results showed that both GA and hydrogen yields were low in the absence of alkali. In the absence of a catalyst, the yields were only slightly higher than those under alkali-free conditions, while no reaction occurred when both alkali and catalyst were absent. This confirms that photocatalysis serves as the primary driving force for xylan depolymerization and subsequent C–C bond cleavage, whereas the alkaline medium provides a source of OH^−^ and assists in glycosidic bond cleavage, but cannot substitute for the role of photocatalysis. Regarding hydrogen production, photogenerated electrons reduce protons on the MCS surface to generate hydrogen, while the oxidation of xylan consumes holes, effectively suppressing electron-hole recombination and ensuring hydrogen evolution efficiency.

Based on the experimental results and previous reports [15,25,75–77,87], a plausible reaction mechanism is proposed as illustrated in Figs. 1 and 6J. Upon the formation of the CNMCS heterostructure, an internal electric field is established from MCS to CN due to the difference in their Fermi levels, which drives charge redistribution at the interface. Under light irradiation, both CN and MCS are photoexcited to generate electron-hole pairs. Following an S-scheme charge transfer pathway, the photogenerated electrons in the CB of CN recombine with the holes in the VB of MCS at the interface. As a result, electrons with strong reduction ability are retained in the CB of MCS, while holes with strong oxidation ability remain in the VB of CN, leading to efficient spatial charge separation. In alkaline media, photogenerated •OH radicals together with OH^−^ effectively cleave the β-1,4-glycosidic bonds, depolymerizing xylan into xylose units [75,85]. Meanwhile, defect sites, amino or hydroxyl functional groups, and the π-conjugated structure on the CNMCS surface facilitate substrate adsorption and activation through hydrogen bonding, n-π* interactions, and C–H/π interactions. Based on this charge distribution, the holes accumulated on the VB of CN can abstract hydrogen atoms from the activated C–H bonds to generate carbon-centered radicals. Simultaneously, the electrons enriched on the CB of MCS reduce O₂ to produce •O_2_^−^ and ^1^O_2_, and also participate in proton reduction to generate hydrogen. Subsequently, the carbon-centered radicals undergo a series of reactions, including retro-aldol cleavage, isomerization, dehydration, and cross-Cannizzaro reactions, ultimately leading to the selective formation of GA [87].

To comprehensively evaluate the environmental compatibility and technical viability of the raw biomass photorefining route, a detailed life cycle assessment (LCA) was conducted. The cradle-to-gate system boundaries were specified for 3 distinct xylan conversion pathways—conventional fermentation, fast pyrolysis, and photocatalytic refining (Figs. S20 to S29). The life cycle inventory (LCI) parameters, comprising material inputs, energy demands, targeted product yields, and secondary emissions, were systematically compiled and validated (Tables S7 to S9). Using the ReCiPe 2016 midpoint methodology, environmental impacts across 18 impact categories were computed (Tables S10 to S13). As illustrated in the comprehensive evaluation (Fig. S26 and Table S13), the photorefining process demonstrates a substantially reduced environmental footprint relative to traditional processing avenues. Notably, regarding global warming potential (GWP) and fossil depletion potential (FDP), photorefining achieved the lowest values.

## Conclusion

In summary, an efficient bifunctional g-C_3_N_4_/Mn_0.7_Cd_0.3_S S-scheme heterojunction photocatalyst (CNMCS) was successfully developed for simultaneous xylan conversion to GA and hydrogen production under sacrificial-agent-free conditions. Structural characterizations confirm the formation of a coral-like heterostructure with intimate interfacial contact between porous g-C_3_N_4_ nanotubes and Mn_0.7_Cd_0.3_S nanoparticles. XPS analysis and DFT calculations further demonstrate the establishment of an S-scheme charge transfer pathway driven by the internal electric field. The optimized 6CNMCS photocatalyst achieves a GA yield of 77.6% and an H_2_ evolution rate of 2.89 mmol•g_cat_^−1^•h^−1^ after 4-h irradiation. In addition, the catalyst retains 85% of the initial GA yield and 91% of the H_2_ evolution activity after 4 cycles, indicating good stability. Mechanistic studies reveal that efficient charge separation and reactive radical generation synergistically promote selective C–C bond cleavage toward GA formation. Crucially, while achieving efficient biomass valorization and hydrogen production, the LCA further reveals that this photorefining technology demonstrates promising sustainability compared to conventional biomass conversion techniques. Overall, this work represents the first demonstration of photocatalytic xylan to GA transformation and offers valuable guidance for modern sustainable biorefineries.

## Methods

### Synthesis of Mn_0.7_Cd_0.3_S nanoparticles (MCS)

Mn_0.7_Cd_0.3_S nanoparticles were prepared by solvothermal methods. The synthesis of Mn_0.7_Cd_0.3_S nanoparticles involved dissolving 0.9375 mmol Cd(CH_3_COO)_2_•2H_2_O, 2.1875 mmol Mn(CH_3_COO)_2_•4H_2_O, and 1.0 g of polyvinylpyrrolidone (PVP) in 25 ml of N,N-dimethylformamide (DMF), followed by 5 min of sonication to form solution A. Separately, solution B was prepared by dissolving 9.375 mmol thioacetamide (TAA) in 25 ml of DMF with the same sonication treatment. After combining solution A with solution B under 10 min of stirring, the mixture was agitated for another 60 min and transferred to a Teflon-lined autoclave. The reaction proceeded at 180 °C for 28 h. The resulting precipitate was thoroughly washed 6 times using alternating anhydrous ethanol and deionized water, and the final product was vacuum-dried at 60 °C overnight, yielding the sample designated as MCS. The Mn_0.7_Cd_0.3_S composition was selected based on our previous systematic study of Mn*_x_*Cd_1-*x*_S solid solutions, in which Mn_0.7_Cd_0.3_S exhibited the highest photocatalytic H_2_ evolution activity among the investigated compositions (Table S6) [15].

### Synthesis of reef-like g-C_3_N_4_ nanotubes (CN)

Urea (10 g) and dicyandiamide (10 g) were mixed by grinding and transferred to a porcelain boat. The boat was then heated to 550 °C at a rate of 5 °C/min in a tube furnace and held for 4 h. Following this calcination step, the temperature was reduced to 500 °C for an additional 2-h annealing. The resulting product was allowed to cool naturally inside the furnace, after which the solid was collected and ground to obtain g-C_3_N_4_ powder, labeled as CN.

### Synthesis of g-C_3_N_4_/Mn_0.7_Cd_0.3_S heterojunctions (CNMCS)

A mixture comprising 0.9375 mmol Cd(CH_3_COO)_2_•2H_2_O, 2.1875 mmol Mn(CH_3_COO)_2_•4H_2_O, 1.0 g of PVP, and a predetermined quantity of CN powder was sonicated in 25 ml of DMF for 5 min to yield a homogeneous solution A. Solution B was prepared by dissolving 9.375 mmol TAA in 25 ml of DMF. Solution A was added to solution B, and a solvent thermal treatment was carried out in a Teflon-lined reactor at 180 °C for 28 h. The samples were dried overnight at 60 °C under a vacuum to obtain 3 wt % g-C_3_N_4_/Mn_0.7_Cd_0.3_S, 6 wt % g-C_3_N_4_/Mn_0.7_Cd_0.3_S, 9 wt % g-C_3_N_4_/Mn_0.7_Cd_0.3_S, 12 wt % g-C_3_N_4_/Mn_0.7_Cd_0.3_S, and 15 wt % g-C_3_N_4_/Mn_0.7_Cd_0.3_S (Fig. 1). The resulting samples were labeled 3CNMCS, 6CNMCS, 9CNMCS, 12CNMCS, and 15CNMCS.

## Data Availability

The data that support the findings of this study are available in the Supplementary Materials.
