# Supplementary material for: Photoconversion of Xylan to Glyceric Acid Coupled with Hydrogen Evolution Enabled by Coral-Like S-Scheme Heterojunction
Source: Research (Wash D C). 2026 Aug 11;9:1399. doi: 10.34133/research.1399 (PMC13457903; doi:10.34133/research.1399)
Supplement: Supplementary file 1 [file research.1399.f1.docx]

*Supplementary Information for*

**Photoconversion of Xylan to Glyceric Acid Coupled with Hydrogen Evolution enabled by Coral-Like S-Scheme Heterojunction**

Yulong An^1,2#^, Yuqi Liu^1,2,3#^, Qiaoge Lu^1,2^, Yiran Zhao^1,2^, Fuyan Kang^1,2^, Yameng Zhang^1,2^, Houjuan Qi^1,2^, Liquan Jing^1,2,4^*, Jinguang Hu^4^*, Zhanhua Huang^1,2^*

^1^State Key Laboratory of Woody Oil Resources Utilization, Northeast Forestry University, Harbin 150040, China

^2^Key Laboratory of Bio-based Material Science and Technology, Ministry of Education, College of Material Science and Engineering, Northeast Forestry University, Harbin 150040, China

^3^School of Environmental Science and Engineering, Tianjin University, Tianjin, 300350, China

^4^Department of Chemical and Petroleum Engineering, University of Calgary, 2500 University Drive, NW, Calgary, Alberta, T2N1N4, Canada

^#^These authors contributed equally to this work.

*Address correspondence to: [liquan.jing@ucalgary.ca](mailto:liquan.jing@ucalgary.ca) (L.J.); [jinguang.hu@ucalgary.ca](mailto:jinguang.hu@ucalgary.ca) (J.H.); [huangzh1975@163.com](mailto:huangzh1975@163.com) (Z.H.)

Number of Pages: 33

Number of Tables: 13

Number of Figures:29

**Table of Contents**

[1. Materials	3](#_Toc235983771)

[2. Characterizations	3](#_Toc235983772)

[3. Experimental details	4](#_Toc235983773)

[4. Theoretical calculations and simulations	5](#_Toc235983774)

[5. Comparison of representative photocatalytic H_2_ evolution systems reported in recent studies.	5](#_Toc235983775)

[6. Morphology of MCS and CN	7](#_Toc235983776)

[7. EPR spectra of CN and CNMCS	7](#_Toc235983777)

[8. Specific surface area and pore size distribution of prepared photocatalysts	8](#_Toc235983778)

[9. Preparation and analysis of glyceric acid calibration curve	9](#_Toc235983779)

[10. Optimization of photocatalytic reaction conditions	9](#_Toc235983780)

[11. Substrate expansion test	12](#_Toc235983781)

[12. Stability analysis of CNMCS catalyst before and after reaction	12](#_Toc235983782)

[13. Comparison with classical methods utilizing xylose and xylan	13](#_Toc235983783)

[14. Light absorption properties of photocatalyst	15](#_Toc235983784)

[15. UPS characterization of CN and MCS	15](#_Toc235983785)

[16. TRPL of photocatalyst	16](#_Toc235983786)

[17. Situ XPS spectra for the 6CNMCS composites	17](#_Toc235983787)

[18. Comparison of the heterojunction mechanisms of CNMCS	18](#_Toc235983788)

[19. CNMCS photocatalytic depolymerization of xylan	19](#_Toc235983789)

[20. DFT calculations for the photocatalysis of xylan over CNMCS	19](#_Toc235983790)

[21. In situ FT-IR spectra of xylan photocatalyzed by CNMCS	21](#_Toc235983791)

[22. Photocatalytic H_2_ evolution performance of Mn_x_Cd_1-x_S solid solutions with different Mn/Cd molar ratios	21](#_Toc235983792)

[23. Sustainability assessment for raw biomass photorefining	22](#_Toc235983793)

# 1. Materials

Benzoquinone (BQ, 99%), Ethylenediaminetetraacetic acid (EDTA, >99.5%), isopropyl (IPA, >99.9%), and L-histidine (99%) were purchased from Aladdin Chemistry Co. (Shanghai, China). Manganese acetate tetrahydrate (Mn(CH_3_COO)_2_·4H_2_O, 99.0%), xylose (99%), and xylan (95%) were purchased from Maclean's Reagent, cadmium acetate dihydrate (Cd(CH_3_COO)_2_·2H_2_O, >99.5%) was purchased from Tianjin Guangfu Fine Chemical Research Institute, thioacetamide (TAA, >99%), polyvinylpyrrolidone (PVP, K30) were purchased from Tianjin Guangfu Science and Technology Development Co. Dimethylformamide (DMF, >99.5%), sodium hydroxide (NaOH, >96%) were purchased from Tianjin Tianli Chemical Reagent Co, glyceric acid (GA, >95%) and dimethyl sulfoxide (DMSO, >99.75%) were obtained from Sinopharm Chemical Reagent Co., Ltd. Urea (>99%) and dicyandiamide (>99%) were purchased from Shanghai Macklin Biochemical Technology Co., Ltd. All the chemicals were of analytical grade without further purification.

# 2. Characterizations

The transmission electron microscope (TEM) observations were performed with a FEI Talos F200X microscope operated at 200 kV (Point resolution 0.25 nm). Transmission electron microscopy (TEM) at 12.5kV (Quanta200, FEI. Netherlands), high resolution transmission electron microscopy (HRTEM) at 200kV acceleration voltage (TEM-2100, LJEMOC, Japan). Scanning Electron Microscopy (SEM) images were obtained by ZEISS Gemini 300. The energy dispersive X-ray spectroscopy (EDX) were conducted on X-ray microanalysis (OXFORD XPLORE30). Photoluminescence (PL, G9800A, Agilent, USA) spectrograms were measured at 375 nm excitation wavelength. X-ray photoelectron spectroscopy (XPS) utilized non-monochromatic Al X-rays as the primary excitation (PHI5700, Thermo Electron Corporation, USA). Fourier transform infrared spectroscopy (FTIR) was obtained by scanning from 4000 to 400 cm^−1^ at a resolution of 4 cm^−1^ (iS10, Nicolet, USA). The powder X-ray diffraction (pXRD) pattern was acquired under Cu Kα irradiation (40 kV, 30 mA) with a scan range of 5-85° and a speed of 5° min^−1^ (XRD-6100, Shimadzu, Japan). The UV-vis diffuse reflectance spectra were obtained by scanning in the range of 200-800 nm on a UV-vis diffuse reflectance spectrometer (UV-vis DRS) (TU-1900, Beijing Purkinje General Instrument Co., Ltd. China) with BaSO_4_ as the reference. The pore size distribution as well as the specific surface area of the catalysts were analyzed using the Brunauer-Emmett-Teller (BET) method on a nitrogen adsorption analyzer (ASAP2020, Micromeritics, USA). Electron spin resonance (ESR) was carried out to capture radicals using 5, 5´-dimethyl-1-pyrroline N-oxide (DMPO), 2, 2, 6, 6-tetramethylpiperidine (TEMP), and 2, 2, 6, 6-tetramethylpiperidinooxy (TEMPO) as spin trap reagents. Xe-lamp (Hxuv300 Visref, Beijing CEJ Tech. Co., Ltd., Beijing, China), gas chromatography (GC-7920, Beijing CEJ Tech. Co., Ltd., Beijing, China) with a thermal conductive detector (TCD). The liquid products were analyzed by high-performance liquid chromatography (HPLC, Agilent 1290 Infinity Ⅱ) with ZORBAX Eclipse Plus C18 (2.1 mm × 50 mm × 1.8 μm) and Agilent Infinity Lab Poroshell 120 HILIC-Z (2.1 mm × 50 mm × 2.7 μm) columns.

# 3. Experimental details

**Experimental procedure:**

Typically, 25 mg of the prepared photocatalyst, 5 g of NaOH, and 0.1 g xylan were dispersed into a 50 mL aqueous solution containing 25 mL water and 25 mL DMSO. The suspension was vacuumed for 0.5 h to remove the existing gas before illumination. The temperature was controlled at 6°C by circulating condensation. Then, the system was irradiated by a 300 W Xe-lamp under magnetic stirring.

**Gas phase product testing:**

The evolved H_2_ gas was automatically quantitatively analyzed every 0.5 h during 4 h photoreforming process by gas chromatography with a thermal conductive detector.

**Liquid phase product testing:**

The liquid phase products were analyzed by high-performance liquid chromatography (HPLC) with a Hi-Plex H (300 mm × 7.7 mm × 8 μm) column. The mobile phase consisted of 5 mM dilute sulfuric acid at a flow rate of 0.6 mL•min^−1^, and the column temperature and detector temperature were set at 60℃. The organic acids were analyzed by a 1290 VWD detector. Ultrapure water was used as the mobile phase, the flow rate was set at 0.6 mL•min^−1^, the column temperature and detector temperature were both set at 45℃, and a 1290 ELSD detector was used to analyze the sugars. The yield of the liquid phase products was calculated according to the conservation of the amount of elemental carbon substances: Yield (%) = (Moles of carbon in the product) / (Moles of carbon in feedstock input) × 100%.

# 4. Theoretical calculations and simulations

**DFT calculations:**

First-principles calculations were carried out on the basis of periodic DFT using a generalized gradient approximation within the Perdew-Burke-Ernzerh of exchange correction functional. The wave functions were constructed from the expansion of plane waves with an energy cutoff of 450 eV. Gamma centered k-point of 3×3×3 or 3×3×1 have been used for geometry optimization for bulk and slab. The consistence tolerances for the geometry optimization are set as 1.0 ×10^−6^ eV/atom for total energy and 0.02 eV/Å for force, respectively. In order to avoid the interaction between the two surfaces, a large vacuum gap of 15 Å has been selected in the periodically repeated slabs.

**Methodology for life cycle assessment (LCA):**

We used LCA for analysis to assess the sustainability of the raw biomass photorefining process. LCA is a systematic quantitative tool for analyzing the environmental impacts of processes, products, and services. This analysis process is conducted under the guidance of International Organization for Standardization (ISO) 14040 and 14044, comprising four stages: goal and scope definition, life cycle inventory (LCI), life cycle impact assessment (LCIA) and interpretation. We conducted the assessment using OpenLCA 2.x software and performed the calculations based on the ecoinvent 3.11 database using the ReCiPe 2016 methodology.

# 5. Comparison of representative photocatalytic H_2_ evolution systems reported in recent studies.

**Table S1.** Comparison of representative photocatalytic H_2_ evolution systems reported in recent studies.

| Photocatalyst | Photocatalytic system | Reaction medium | Light Source | Main reaction | H_2_ (mmol•g_cat_^−1^•h^−1^) | | Ref. |
| --- | --- | --- | --- | --- | --- | --- | --- |
| CuInS_2_ QD-g-C_3_N_4_  (20 mg) | 80 mL deionized water | 10 mL TEOA | visible light | Water splitting | 0.10 | [50] | |
| Ni@TiO_2_/ g-C_3_N_4_  (50 mg) | 150 mL water solution | 10 % TEOA | visible light | Water splitting | 0.13 | [51] | |
| CdS/NiCoAl-LDH  (20 mg) | 50 mL deionized water | Na_2_S (0.25 M) and Na_2_SO_3_ (0.35 M) | visible light | Water splitting | 2.12 | [52] | |
| CdS@Ce-MOF/g-C_3_N_4_  (20 mg) | 50 mL water, 1 % Pt | 10 vol% TEOA | visible light | Water splitting | 0.83 | [53] | |
| polymeric carbon nitride  (20  mg) | 45 mL water, 3 wt% Pt | 5 mL benzyl alcohol | Dark reaction after 1 h of visible light exposure | Water splitting and oxidation of benzyl alcohol | 1.48 | [54] | |
| Benzene Rings Doped g-C_3_N_4_  (50 mg) | 100 mL water, 1 wt% Pt | 10 vol% TEOA | visible light | Water splitting | 0.52 | [55] | |
| g-C_3_N_4_  (50 mg) | 90 ml water, 3 wt.% Pt | 10 mL TEOA | visible light | Water splitting | 2.66 | [56] | |
| CN/CNQDs-20-6 h  (20 mg) | 50 mL water, 2.0 wt% Pt | 10 vol% TEOA | visible light | Water splitting | 1.09 | [57] | |
| g-C_3_N_4_/  Mn_0.7_Cd_0.3_S  (25 mg) | 25 mL water and 25 mL DMSO | xylan | visible light | Water splitting and photoreforming of xylan | 2.89 | This work | |

As summarized in Table S1, previously reported g-C_3_N_4_-based photocatalytic systems generally rely on costly noble-metal co-catalysts (e.g., 1-3 wt% Pt) or external sacrificial electron donors (e.g., TEOA, Na_2_S/Na_2_SO_3_), yielding H_2_ evolution rates between 0.10 and 2.66 mmol•g_cat_^−1^•h^−1^. Although systems such as Pt/g-C_3_N_4_ (2.66 mmol•g_cat_^−1^•h^−1^) and Pt/polymeric carbon nitride (1.48 mmol•g_cat_^−1^•h^−1^) exhibit high activity, the scarcity of Pt and the secondary pollution from sacrificial agents limit their practical viability. In contrast, our noble-metal-free g-C_3_N_4_/Mn_0.7_Cd_0.3_S heterojunction achieves a higher H_2_ evolution rate of 2.89 mmol•g_cat_^−1^•h^−1^ under visible light without any added sacrificial agents, using xylan itself as the electron donor. This activity stems from the built-in electric field across the S-scheme interface: the IEF promotes the selective recombination of weaker electrons and holes while retaining highly reductive conduction-band electrons on Mn_0.7_Cd_0.3_S to drive proton reduction.

# 6. Morphology of MCS and CN

**Fig. S1.** SEM images of (a, b) MCS and (c) CN.

# 7. EPR spectra of CN and CNMCS

**Fig. S2.** EPR spectra of CN and CNMCS.

# 8. Specific surface area and pore size distribution of prepared photocatalysts

**Fig. S3.** corresponding pore size distributions of the CN, 6CNMCS, and MCS.

**Table S2.** The specific surface area and pore size of CN, CNMCS, and MCS.

| Photocatalysts | Specific surface area  (m^2^•g^-1^) | Pore size  (nm) |
| --- | --- | --- |
| CN | 13.0563 | 6-60 |
| 3CNMCS | 17.6253 | 10-26 |
| 6CNMCS | 19.7507 | 17-39 |
| 9CNMCS | 15.4910 | 14-27 |
| 12CNMCS | 11.6855 | 14-27 |
| 15CNMCS | 10.9943 | 15-27 |
| MCS | 23.1671 | 23-52 |

# 9. Preparation and analysis of glyceric acid calibration curve

**Fig. S4.** Calibration curve of glyceric acid.

A glyceric acid standard (purity ≥ 95%, purchased from Sinopharm Chemical Reagent Co., Ltd.) was used to prepare a series of standard aqueous solutions with concentrations of 0.01, 0.05, 0.10, 0.25, and 0.50 g•L^-1^. The standard solutions were analyzed under the same HPLC conditions as the reaction samples.

The calibration curve was established by plotting the chromatographic peak area against glyceric acid concentration, showing excellent linearity with a correlation coefficient (R^2^) greater than 0.999. The concentration of glyceric acid in the samples was quantified according to the calibration curve and corrected using the corresponding dilution factors.

# 10. Optimization of photocatalytic reaction conditions

Owing to the complex process of photocatalytic reactions, the photocatalytic performance is not only related to the band structure, light absorption properties, and electrochemical properties of the materials, but also subject to the conditions of light intensity, pH of the reaction system, substrate concentration, solvent polarity, and catalyst dosage. Because of the polymer properties of xylan and the powder character of the CNMCS used in the experiments, the NaOH dosage, xylan dosage, and catalyst dosage were selected as the variables to optimize the photocatalytic performance to increase the rate of H_2_ production and glyceric acid yield.

**Fig. S5.** Effect of NaOH dosage on the production of (a) H_2_ and (b) glyceric acid from photoreforming xylan

As shown in Fig. S5a-b, the dosage of NaOH has a strongly influencing effect on the photoreforming xylan reaction, and the rate of H_2_ evolution is significantly increased with the increase of NaOH dosage, which is due to the reason that •OH promotes the H_2_ production half-reaction through the redox shuttle mechanism in the presence of alkali. However, when the alkali concentration is too high, the surface of the CdS-based materials will appear a layer of dense oxide film, which will cause the rate of the reaction to decrease [50]. A high concentration of substrate in the photoreforming reaction can lead to a slowing down of the adsorption and desorption processes of the feedstock and products, which will affect the reaction. However, when the substrate in the system is low, the rate of the biomass redox half-reaction is slower, which in turn affects the H_2_ production half-reaction and ultimately leads to a lower overall reaction rate (Fig. S6a-b) [17]. Similarly, the photocatalytic performance is also influenced by catalyst dosage (Fig. S7a-b). As the catalyst amount increased from 5 to 25 mg, both H_2_ evolution and glyceric acid production gradually increased because more active sites became available. However, excessive catalyst loading (35 mg) slightly decreased the activity, which may be attributed to enhanced light scattering and reduced light penetration in the suspension system. The optimal dosage of NaOH, xylan, and catalyst used in the 6CNMCS photoreforming xylan reaction is 5.0 g, 100 mg, and 25 mg, which corresponds to an H_2_ production rate and glyceric acid yield of 2.89 mmol•g_cat_^−1^•h^−1^ and 77.6%, respectively. The photocatalytic reaction will be carried out under optimal conditions.

**Fig. S6.** Effect of xylan dosage on the production of (a) H_2_ and (b) glyceric acid from photoreforming xylan

**Fig. S7.** Effect of photocatalyst dosage on the production of (a) H_2_ and (b) glyceric acid from photoreforming xylan

The optimized NaOH loading of 5.0 g (2.5 M) is necessary to create a strongly alkaline environment (pH ≈ 13), which promotes xylan dissolution and hydrolysis, exposes more active sites, and stabilizes glyceric acid in its salt form to prevent over-oxidation or polymerization on the catalyst surface. From a scalability perspective, the use of 2.5 M NaOH may increase chemical consumption and wastewater treatment requirements. However, high-concentration alkaline systems are commonly employed in lignocellulosic biorefinery processes, such as alkaline pretreatment and kraft pulping, where mature technologies for neutralization, membrane separation, and chemical recovery are routinely applied. Therefore, although 5.0 g of NaOH per batch is relatively high at the laboratory scale, it is technically feasible for industrial processing, especially considering the high added value of glyceric acid and H_2_. The environmental and economic impacts of NaOH consumption can be further mitigated through alkali recovery and recycling. After product separation (e.g., acidification, extraction, or electrodialysis), the residual alkaline reaction solution still contains a substantial amount of NaOH, which could be reused in subsequent batches, thereby reducing overall alkali consumption and environmental impact.

# 11. Substrate expansion test

**Fig. S8.** The (a) H_2_ production and (b) glyceric acid yield results of photocatalytic reactions using xylose, arabinose, glucose, fructose, xylan, and dextran as substrates, respectively.

# 12. Stability analysis of CNMCS catalyst before and after reaction

To further investigate the structural stability of the catalyst after photoreforming, the recovered 6CNMCS catalyst was characterized by XRD and XPS. As shown in Fig. S9, the XRD patterns before and after reaction remain largely unchanged, with no impurity peaks or significant phase transformation observed. This indicates that the crystalline structure of the CNMCS heterostructure is well preserved during photocatalysis. XPS spectra of the recovered catalyst (Fig. S10) show that the characteristic peaks of C, N, Mn, Cd, S, and O are maintained, with negligible binding-energy shifts compared with the fresh catalyst. In particular, the Cd 3d and S 2p spectra exhibit minimal changes, suggesting that photocorrosion of MnCdS is effectively suppressed.

ICP-MS analysis of the post-reaction solution reveals a slight Cd^2+^ leaching concentration of 0.81 mg•L^-1^, corresponding to only 0.49% of the total Cd initially present in 6CNMCS. The low dissolution ratio further demonstrates the strong structural stability and resistance of the heterostructure to photocorrosion during the photoreforming reaction.

**Fig. S9.** XRD patterns showing crystal structure.

**Fig. S10.** XPS spectra of 6CNMCS composite material before and after photocatalytic reaction: (a) C 1s, (b) N 1s, (c) Mn 2p, (d) Cd 3d, (e) S 2p.

# 13. Comparison with classical methods utilizing xylose and xylan

**Table S3.** Comparison of several representative methods for the production of high value-added chemicals from xylose and xylan.

| Substrates | Methods | H_2_ evolution rate (mmol•g_cat_^−1^•h^−1^) | Products | Yields  (%) | Ref. |
| --- | --- | --- | --- | --- | --- |
| Xylan | Pyrolysis | – | Furfural | 16.5 | [75] |
| Xylan | Fermentation | – | Xylitol | 66.0 | [77] |
| Xylan | Solid-acid catalysis | – | Xylose | 74.1 | [78] |
| Xylose | Photocatalysis | 7.59 | Xylonic acid | 56.9 | [79] |
| Xylose | Photocatalysis | 14.06 | Glyceric acid | 38.2 | [17] |
| Xylan | Photocatalysis | 2.89 | Glyceric acid | 77.6 | This work |

Table S3 compares the operational characteristics and product distributions across thermochemical, biological, and photocatalytic pathways for upgrading xylose and xylan. Thermochemical pyrolysis yields furfural from xylan at only 16.5% due to unselective thermal cracking. Fermentation and solid-acid catalysis offer moderate yields of xylitol (66.0%) or xylose (74.1%), but these routes require intensive pretreatments and tight operating control, without co-generating clean H_2_ energy. In photocatalytic systems, while monosaccharides such as xylose can achieve high H_2_ evolution rates (e.g., 14.06 mmol•g_cat_^−1^•h^−1^), the unmoderated oxidation potentials often trigger non-selective over-oxidation to CO_2_ or short-chain organic acids, limiting the yield of target C3 molecules like glyceric acid to 38.2%. Directly photoreforming raw polymeric xylan is considerably more challenging due to its resistant β-1,4-glycosidic linkages. In our g-C_3_N_4_/Mn_0.7_Cd_0.3_S system, the S-scheme architecture moderates the oxidation capability at the g-C_3_N_4_ domain, which selectively drives C-H/C-C bond cleavage in xylan while suppressing full mineralization. As a result, our system delivers a 77.6% yield of glyceric acid alongside a steady H_2_ evolution rate of 2.89 mmol•g_cat_^−1^•h^−1^, achieving high target-product selectivity directly from macromolecular biomass.

# 14. Light absorption properties of photocatalyst

**Fig. S11.** UV-Vis DRS of CN, CNMCS, and MCS.

# 15. UPS characterization of CN and MCS

**Fig. S12**. UPS spectra of (a) CN and (b) MCS.

To experimentally determine the work function (*Φ*) of the photocatalysts, UPS measurements were performed using a He I (*hν* = 21.22 eV) excitation source. The secondary electron cutoff (E_cutoff_) and Fermi edge (E_Fermi_) were identified, and the work function was calculated using *Φ* = *hν* − (E_cutoff_ − E_Fermi_).

As shown in Fig. S12, the secondary electron cutoff for CN and MCS were determined to be 16.68 eV and 17.12 eV, respectively, with corresponding Fermi levels at 0 eV. Based on these values, the work functions of CN and MCS were calculated to be 4.54 eV and 4.10 eV, respectively. These results are in close agreement with the DFT-calculated values (4.61 eV for CN and 4.19 eV for MCS), confirming that CN possesses a higher work function than MCS.

This difference in work function experimentally supports the proposed S-scheme charge transfer mechanism: upon intimate contact in the CNMCS heterojunction, electrons preferentially transfer from MCS (lower *Φ*) to CN (higher *Φ*) until Fermi level equilibration occurs. This establishes an internal electric field directed from MCS to CN, which drives selective recombination at the interface and preserves strongly reductive electrons in the CB of MCS and strongly oxidative holes in the VB of CN, enabling efficient hydrogen evolution and xylan oxidation.

# 16. TRPL of photocatalyst

**Table S4.** The fitting parameters of TR-PL decay curves according to bi-exponential function.

| Sample | τ_1_ (ns) | τ_2_ (ns) | A_1_ (%) | A_2_ (%) | τ_a_ (ns)*^a^* |
| --- | --- | --- | --- | --- | --- |
| MCS | 2.25 | 2.85 | 12.61 | 32.58 | 2.71 |
| CN | 1.67 | 2.71 | 78.23 | 14.55 | 1.91 |
| CNMCS | 1.38 | 2.32 | 138.97 | 10.72 | 1.49 |

*^a^* The average recombination lifetimes (τ_a_) were calculated by the equation τ_a._= (A_1_τ_1_^2^ + A_2_τ_2_^2^) / (A_1_τ_1_ + A_2_τ_2_).

To further elucidate the influence of the heterogeneous interface on carrier dynamics, TR-PL decay measurements were performed. The TR-PL decay curves were fitted using a biexponential function: *I*(t) = *A*_1_ exp(−*t*/τ_1_) + *A*_2_ exp(−*t*/τ_2_), where τ₁ and τ₂ represent the recombination lifetimes, and *A*_1_ and *A*_2_ correspond to their respective amplitudes.

The fast decay component (τ_1_, *A*_1_) is attributed to surface nonradiative recombination, while the slow decay component (τ_2_, *A*_2_) originates from bulk radiative recombination. Compared with MCS and CN, CNMCS exhibits smaller τ_2_ and *A*_2_ values (Table S4), indicating that the heterostructure significantly enhances the bulk transfer and separation of photogenerated charge carriers. Based on the biexponential fitting, the average recombination lifetimes (τ_a_) of MCS, CN, and CNMCS were calculated to be 2.71, 1.91, and 1.49 ns, respectively. The shortest τ_a_ further demonstrates that both bulk and surface charge recombination are effectively suppressed in CNMCS. Instead of undergoing recombination, the photoexcited carriers are rapidly funneled into nonradiative pathways associated with interfacial charge transfer.

# 17. Situ XPS spectra for the 6CNMCS composites

**Fig. S13**. In situ XPS spectra for the 6CNMCS composites: (a) C 1s, (b) N 1s, (c) Mn 2p, (d) Cd 3d, (e) S 2p.

# 18. Comparison of the heterojunction mechanisms of CNMCS

**Table S5**. Comparison of charge-transfer characteristics between Type-II, conventional Z-scheme, and S-scheme heterojunction mechanisms.

| Mechanism | Charge transfer pathway | Redox ability | Band bending / IEF | Expected XPS shift under illumination | Consistency with CNMCS results |
| --- | --- | --- | --- | --- | --- |
| Type-II heterojunction | Electrons migrate from CN CB to MCS CB; holes migrate from MCS VB to CN VB | Oxidation and reduction abilities are weakened due to carrier transfer to lower-energy bands | Weak interfacial electric field | Simultaneous electron accumulation on one component | Inconsistent |
| Conventional Z-scheme heterojunction | Electrons in MCS CB recombine with holes in CN VB through mediator/interface | Strong redox ability retained | Interfacial recombination pathway required | Charge-transfer direction opposite to observed XPS shifts | Partially consistent |
| S-scheme heterojunction | Electrons in CN CB recombine with holes in MCS VB driven by band bending and IEF | Strong oxidation and reduction abilities preserved | Built-in electric field and asymmetric band bending | Positive XPS shifts for CN and negative shifts for MCS under illumination | Highly consistent |

The comparison further supports that the charge-transfer behavior observed in the CNMCS heterojunction is more consistent with the S-scheme mechanism than with conventional Type-II or Z-scheme pathways.

# 19. CNMCS photocatalytic depolymerization of xylan

**Fig. S14**. Photocatalytic depolymerization of xylan into xylose and xylobiose under control conditions: without light (No Light), without alkali (No CAT), and without both alkali and light (No ALK + Light). (Reaction conditions: xylan: 100 mg, CNMCS: 25 mg, NaOH solution: 2.5M, 50mL irradiation time: 30 min, reaction temperature: 6 °C)

# 20. DFT calculations for the photocatalysis of xylan over CNMCS

**Fig. S15**. (a) Top view and (b) side view of the optimized structure of CN.

**Fig. S16**. (a) Top view and (b) side view of the optimized structure of CNMCS.

**Fig. S17**. Optimized geometric structures of xylose adsorbed on (a) CN and (b) CNMCS surfaces.

**Fig. S18**. The optimized geometries for the conversion of xylose to glyceric acid on CNMCS.

# 21. In situ FT-IR spectra of xylan photocatalyzed by CNMCS

**Fig. S19**. In-situ FT-IR spectra of CNMCS for the adsorption of xylan in the dark and under light illumination for another 1 h.

# 22. Photocatalytic H_2_ evolution performance of Mn_x_Cd_1-x_S solid solutions with different Mn/Cd molar ratios

**Table S6.** Photocatalytic H_2_ evolution performance of Mn_x_Cd_1-x_S solid solutions with different Mn/Cd molar ratios reported in our previous work [15].

| Mn_x_Cd_1-x_S | Mn/Cd molar ratio | H_2_ evolution  (mmol•g^-1^•h^-1^) |
| --- | --- | --- |
| Mn_1_Cd_9_S | 1:9 | 0.57 |
| Mn_3_Cd_7_S | 3:7 | 2.28 |
| Mn_5_Cd_5_S | 5:5 | 4.03 |
| Mn_7_Cd_3_S | 7:3 | 14.06 |
| Mn_9_Cd_1_S | 9:1 | 1.18 |

# 23. Sustainability assessment for raw biomass photorefining

**Table S7.** Material and energy inventory data of fermentation (based on processing 1 kg of xylan).

| Unit process | Material | Value | Units |
| --- | --- | --- | --- |
| Pyrolysis | Input | | |
|  | Xylan (F1) | 1 | Kg |
|  | K_2_HPO_4_ (F2) | 0.2 | kg |
|  | NH_4_NO_3_ (F3) | 0.2 | kg |
|  | MgSO_4_•7H_2_O (F4) | 0.02 | kg |
|  | Deionized water (F5) | 100 | L |
|  | Fermentation broth (F6) | 0.05 | kg |
|  | Electricity (F7) | 17.3 | kWh |
| Product | Output | | |
|  | 2,3-butanediol | 0.389 | kg |
|  | Water | 100 | mL |
|  | Waste gas | 0.3 | kg |
|  | Solid waste | 0.05 | kg |

**Table S8.** Material and energy inventory data of pyrolysis (based on processing 1 kg of xylan).

| Unit process | Material | Value | Units |
| --- | --- | --- | --- |
| Pyrolysis | Input | | |
|  | Xylan (P1) | 1 | Kg |
|  | N_2_ (P2) | 0.0124 | kg |
|  | Deionized water (P3) | 10 | kg |
|  | Electricity (P4) | 12.3 | kWh |
| Product | Output | | |
|  | CO, CO_2_, CH_4_ | 0.27 | Kg |
|  | Water | 0.231 | Kg |
|  | Bio-oil | 0.306 | Kg |
|  | Solid waste | 0.193 | Kg |
|  | Waste gas | 0.0037 | kg |

**Table S9.** Material and energy inventory data of photorefining (based on processing 1 kg of xylan).

| Unit process | Material | Value | Units |
| --- | --- | --- | --- |
| Pyrolysis | Input | | |
|  | Urea (A1) | 0.00225 | kg |
|  | Dicyandiamide (A2) | 0.00225 | kg |
|  | (CH_3_COO)_2_Cd (A3) | 0.01743 | kg |
|  | (CH_3_COO)_2_Mn (A4) | 0.0374 | kg |
|  | PVP (A5) | 0.025 | kg |
|  | TAA (A6) | 0.01638 | kg |
|  | DMF (A7) | 0.6 | kg |
|  | Deionized water 1 (A8) | 0.5 | kg |
|  | ethyl alcohol (A9) | 0.2 | kg |
|  | Electricity 1 (A10) | 0.02 | Kwh |
|  | NaOH (A11) | 0.8 | kg |
|  | Xylan (A12) | 1 | kg |
|  | Deionized water 2 (A13) | 0.25 | kg |
|  | DMSO (A14) | 0.25 | kg |
|  | Electricity 2 (A15) | 2.25 | kWh |
| Product | Output | | |
|  | Glyceric acid | 0.776 | kg |
|  | H_2_ | 2.89 | mmol |
|  | Water | 10 | kg |
|  | Abandoned catalysts | 0.25 | kg |

**Table S10.** Evaluation of the effects of fermentation for xylan on the production of platform compound.

| Impact category | Unit | Impact scores of each subprocess of conventional fermentation | | | | | | |
| --- | --- | --- | --- | --- | --- | --- | --- | --- |
|  |  | Xylan | K_2_HPO_4_ | NH_4_NO_3_ | MgSO_4_•7H_2_O | Deionized water | fermentation broth | Electricity |
| Global warming (GWP) | kg CO_2_ eq | 6.1155 | 0.5387 | 1.7290 | 0.0092 | 0.0584 | 0.1770 | 22.7093 |
| Stratospheric ozone depletion (SOD) | kg CFC_11_ eq | 0.0000 | 0.0000 | 0.0000 | 0.0000 | 0.0000 | 0.0000 | 0.0000 |
| Ionizing radiation (IR) | kBq Co-60 eq | 0.2221 | 0.0377 | 0.0216 | 0.0013 | 0.0349 | 0.0035 | 0.0789 |
| Ozone formation, Human health (OFH) | kg NO_x_ eq | 0.0155 | 0.0014 | 0.0031 | 0.0000 | 0.0001 | 0.0002 | 0.0665 |
| Fine particulate matter formation (FPM) | kg PM_2.5_ eq | 0.0146 | 0.0015 | 0.0017 | 0.0000 | 0.0001 | 0.0002 | 0.0455 |
| Ozone formation, Terrestrial ecosystems (OFT) | kg NO_x_ eq | 0.0157 | 0.0015 | 0.0031 | 0.0000 | 0.0001 | 0.0003 | 0.0667 |
| Terrestrial acidification (TA) | kg SO_2_ eq | 0.0353 | 0.0035 | 0.0061 | 0.0000 | 0.0003 | 0.0008 | 0.1335 |
| Freshwater eutrophication (FE) | kg P eq | 0.0038 | 0.0003 | 0.0002 | 0.0000 | 0.0000 | 0.0000 | 0.0126 |
| Marine eutrophication (ME) | kg N eq | 0.0003 | 0.0000 | 0.0000 | 0.0000 | 0.0000 | 0.0002 | 0.0008 |
| Terrestrial ecotoxicity (TE) | kg 1,4-DCB | 33.9241 | 2.2664 | 4.6557 | 0.0149 | 0.2444 | 0.3026 | 11.4423 |
| Freshwater ecotoxicity (FEO) | kg 1,4-DCB | 0.3118 | 0.0222 | 0.0292 | 0.0002 | 0.0030 | 0.0028 | 0.4373 |
| Marine ecotoxicity (MEO) | kg 1,4-DCB | 0.4476 | 0.0319 | 0.0435 | 0.0003 | 0.0042 | 0.0036 | 0.6107 |
| Human carcinogenic toxicity (HCT) | kg 1,4-DCB | 0.3581 | 0.0226 | 0.0212 | 0.0004 | 0.0143 | 0.0031 | 1.1223 |
| Human non-carcinogenic toxicity (HNC) | kg 1,4-DCB | 10.2946 | 0.7380 | 1.0161 | 0.0048 | 0.0748 | 0.0411 | 12.3549 |
| Land use (LU) | m^2^a crop eq | 0.3187 | 0.0559 | 0.0109 | 0.0002 | 0.0021 | 0.1014 | 0.2245 |
| Mineral resource scarcity (MRS) | kg Cu eq | 0.0335 | 0.0107 | 0.0074 | 0.0000 | 0.0006 | 0.0003 | 0.0048 |
| Fossil resource scarcity (FDP) | kg oil eq | 1.5584 | 0.1693 | 0.2449 | 0.0024 | 0.0152 | 0.0205 | 5.4964 |
| Water consumption (WC) | m^3^ | 0.3729 | 0.0142 | 0.0153 | 0.0001 | 0.1035 | 0.0028 | 0.0378 |

**Fig. S20**. LCA of fermentation: GWP and FDP scores. (The abbreviations represent the names listed in Table S7).

**Fig. S21**. Environmental scoring of process variables. Environmental scores for process variables in fermentation technology for xylan. (The abbreviations represent the names listed in Table S7).

**Table S11.** Evaluation of the effects of pyrolysis for xylan on the production of platform compound.

| Impact category | Unit | Impact scores of each subprocess of conventional pyrolysis | | | |
| --- | --- | --- | --- | --- | --- |
|  |  | Xylan | N_2_ | Deionized water | Electricity |
| Global warming (GWP) | kg CO_2_ eq | 6.1155 | 0.0066 | 0.0058 | 16.1459 |
| Stratospheric ozone depletion (SOD) | kg CFC_11_ eq | 0.0000 | 0.0000 | 0.0000 | 0.0000 |
| Ionizing radiation (IR) | kBq Co-60 eq | 0.2221 | 0.0169 | 0.0035 | 0.0561 |
| Ozone formation, Human health (OFH) | kg NO_x_ eq | 0.0155 | 0.0000 | 0.0000 | 0.0472 |
| Fine particulate matter formation (FPM) | kg PM_2.5_ eq | 0.0146 | 0.0000 | 0.0000 | 0.0323 |
| Ozone formation, Terrestrial ecosystems (OFT) | kg NO_x_ eq | 0.0157 | 0.0000 | 0.0000 | 0.0474 |
| Terrestrial acidification (TA) | kg SO_2_ eq | 0.0353 | 0.0000 | 0.0000 | 0.0949 |
| Freshwater eutrophication (FE) | kg P eq | 0.0038 | 0.0000 | 0.0000 | 0.0089 |
| Marine eutrophication (ME) | kg N eq | 0.0003 | 0.0000 | 0.0000 | 0.0005 |
| Terrestrial ecotoxicity (TE) | kg 1,4-DCB | 33.9241 | 0.0323 | 0.0244 | 8.1352 |
| Freshwater ecotoxicity (FEO) | kg 1,4-DCB | 0.3118 | 0.0004 | 0.0003 | 0.3109 |
| Marine ecotoxicity (MEO) | kg 1,4-DCB | 0.4476 | 0.0005 | 0.0004 | 0.4342 |
| Human carcinogenic toxicity (HCT) | kg 1,4-DCB | 0.3581 | 0.0004 | 0.0014 | 0.7979 |
| Human non-carcinogenic toxicity (HNC) | kg 1,4-DCB | 10.2946 | 0.0124 | 0.0075 | 8.7841 |
| Land use (LU) | m^2^a crop eq | 0.3187 | 0.0073 | 0.0002 | 0.1596 |
| Mineral resource scarcity (MRS) | kg Cu eq | 0.0335 | 0.0001 | 0.0001 | 0.0034 |
| Fossil resource scarcity (FDP) | kg oil eq | 1.5584 | 0.0018 | 0.0015 | 3.9079 |
| Water consumption (WC) | m^3^ | 0.3729 | 0.0009 | 0.0104 | 0.0268 |

**Fig. S22**. LCA of pyrolysis: GWP and FDP Scores (The abbreviations represent the names listed in Table S8).

**Fig. S23**. Environmental scoring of process variables. Environmental scores for process variables in pyrolysis technology for xylan. (The abbreviations represent the names listed in Table S8).

**Table S12.** Evaluation of the effects of photorefining for xylan on the production of glyceric acid.

| Impact category | Unit | | Impact scores of each subprocess of photorefining | | | | | | | | | | | | | | | | | | | | | | | | | | | | | |
| --- | --- | --- | --- | --- | --- | --- | --- | --- | --- | --- | --- | --- | --- | --- | --- | --- | --- | --- | --- | --- | --- | --- | --- | --- | --- | --- | --- | --- | --- | --- | --- | --- |
|  |  |  | urea | | dicyandiamide | | (CH_3_COO)_2_Cd | | (CH_3_COO)_2_Mn | | PVP | | TAA | | DMF | | Deionized water 1 | | ethyl alcohol | | NaOH | | Xylan | | Deionized water 2 | | DMSO | | | Electricity 1 | Electricity 2 |  |
| Global warming (GWP) | kg CO_2_ eq | | 0.0259 | | 0.0312 | | 0.0362 | | 0.1107 | | 0.0505 | | 0.0615 | | 1.7013 | | 0.0003 | | 0.1730 | | 1.1047 | | 6.1155 | | 0.0001 | | 0.3292 | | | 0.0263 | 2.9535 |  |
| Stratospheric ozone depletion (SOD) | kg CFC_11_ eq | | 0.0000 | | 0.0000 | | 0.0000 | | 0.0000 | | 0.0000 | | 0.0000 | | 0.0000 | | 0.0000 | | 0.0000 | | 0.0000 | | 0.0000 | | 0.0000 | | 0.0000 | | | 0.0000 | 0.0000 |  |
| Ionizing radiation (IR) | kBq Co-60 eq | | 0.0018 | | 0.0024 | | 0.0016 | | 0.0156 | | 0.0017 | | 0.0015 | | 0.1236 | | 0.0002 | | 0.0066 | | 0.1284 | | 0.2221 | | 0.0001 | | 0.0362 | | | 0.0001 | 0.0103 |  |
| Ozone formation, Human health (OFH) | kg NO_x_ eq | | 0.0001 | | 0.0001 | | 0.0001 | | 0.0002 | | 0.0001 | | 0.0001 | | 0.0034 | | 0.0000 | | 0.0004 | | 0.0028 | | 0.0155 | | 0.0000 | | 0.0006 | | | 0.0001 | 0.0086 |  |
| Fine particulate matter formation (FPM) | kg PM_2.5_ eq | | 0.0001 | | 0.0000 | | 0.0001 | | 0.0002 | | 0.0000 | | 0.0001 | | 0.0027 | | 0.0000 | | 0.0003 | | 0.0025 | | 0.0146 | | 0.0000 | | 0.0004 | | | 0.0001 | 0.0059 |  |
| Ozone formation, Terrestrial ecosystems (OFT) | kg NO_x_ eq | | 0.0001 | | 0.0001 | | 0.0001 | | 0.0003 | | 0.0001 | | 0.0001 | | 0.0036 | | 0.0000 | | 0.0004 | | 0.0028 | | 0.0157 | | 0.0000 | | 0.0007 | | | 0.0001 | 0.0087 |  |
| Terrestrial acidification (TA) | kg SO_2_ eq | | 0.0002 | | 0.0001 | | 0.0002 | | 0.0004 | | 0.0001 | | 0.0003 | | 0.0063 | | 0.0000 | | 0.0006 | | 0.0043 | | 0.0353 | | 0.0000 | | 0.0011 | | | 0.0002 | 0.0174 |  |
| Freshwater eutrophication (FE) | kg P eq | | 0.0000 | | 0.0000 | | 0.0000 | | 0.0001 | | 0.0000 | | 0.0000 | | 0.0006 | | 0.0000 | | 0.0000 | | 0.0006 | | 0.0038 | | 0.0000 | | 0.0001 | | | 0.0000 | 0.0016 |  |
| Impact category | | Unit | | Impact scores of each subprocess of photorefining | | | | | | | | | | | | | | | | | | | | | | | | | | | | |
|  |  |  |  | urea | | dicyandiamide | | (CH_3_COO)_2_Cd | | (CH_3_COO)_2_Mn | | PVP | | TAA | | DMF | | Deionized water 1 | | ethyl alcohol | | NaOH | | Xylan | | Deionized water 2 | | DMSO | Electricity 1 | | Electricity 2 |  |
| Marine eutrophication (ME) | | kg N eq | | 0.0000 | | 0.0000 | | 0.0000 | | 0.0000 | | 0.0000 | | 0.0000 | | 0.0018 | | 0.0000 | | 0.0000 | | 0.0001 | | 0.0003 | | 0.0000 | | 0.0000 | 0.0000 | | 0.0001 |  |
| Terrestrial ecotoxicity (TE) | | kg 1,4-DCB | | 0.0820 | | 0.0526 | | 0.0980 | | 0.3497 | | 0.0162 | | 0.1107 | | 5.9876 | | 0.0012 | | 0.1738 | | 3.2757 | | 33.9241 | | 0.0006 | | 0.9370 | 0.0132 | | 1.4882 |  |
| Freshwater ecotoxicity (FEO) | | kg 1,4-DCB | | 0.0009 | | 0.0006 | | 0.0013 | | 0.0035 | | 0.0001 | | 0.0009 | | 0.0488 | | 0.0000 | | 0.0021 | | 0.0389 | | 0.3118 | | 0.0000 | | 0.0117 | 0.0005 | | 0.0569 |  |
| Marine ecotoxicity (MEO) | | kg 1,4-DCB | | 0.0012 | | 0.0009 | | 0.0019 | | 0.0049 | | 0.0001 | | 0.0013 | | 0.0680 | | 0.0000 | | 0.0031 | | 0.0545 | | 0.4476 | | 0.0000 | | 0.0168 | 0.0007 | | 0.0794 |  |
| Human carcinogenic toxicity (HCT) | | kg 1,4-DCB | | 0.0010 | | 0.0009 | | 0.0018 | | 0.0040 | | 0.0002 | | 0.0014 | | 0.0528 | | 0.0001 | | 0.0049 | | 0.0533 | | 0.3581 | | 0.0000 | | 0.0112 | 0.0013 | | 0.1460 |  |
| Human non-carcinogenic toxicity (HNC) | | kg 1,4-DCB | | 0.0252 | | 0.0196 | | 0.0408 | | 0.1032 | | 0.0026 | | 0.0266 | | 1.4524 | | 0.0004 | | 0.0559 | | 1.1127 | | 10.2946 | | 0.0002 | | 0.3734 | 0.0143 | | 1.6068 |  |
| Land use (LU) | | m^2^a crop eq | | 0.0005 | | 0.0019 | | 0.0028 | | 0.0029 | | 0.0006 | | 0.0005 | | 0.0273 | | 0.0000 | | 0.0023 | | 0.0229 | | 0.3187 | | 0.0000 | | 0.0059 | 0.0003 | | 0.0292 |  |
| Mineral resource scarcity (MRS) | | kg Cu eq | | 0.0001 | | 0.0001 | | 0.0001 | | 0.0022 | | 0.0000 | | 0.0001 | | 0.0050 | | 0.0000 | | 0.0003 | | 0.0032 | | 0.0335 | | 0.0000 | | 0.0013 | 0.0000 | | 0.0006 |  |
| Fossil resource scarcity (FDP) | | kg oil eq | | 0.0083 | | 0.0060 | | 0.0146 | | 0.0449 | | 0.0278 | | 0.0340 | | 0.9865 | | 0.0001 | | 0.1946 | | 0.2787 | | 1.5584 | | 0.0000 | | 0.2998 | 0.0064 | | 0.7149 |  |
| Water consumption (WC) | | m^3^ | | 0.0005 | | 0.0003 | | 0.0007 | | 0.0027 | | 0.0049 | | 0.0008 | | 0.0239 | | 0.0005 | | 0.0004 | | 0.0280 | | 0.3729 | | 0.0003 | | 0.0068 | 0.0000 | | 0.0049 |  |

**Fig. S24**. LCA of photorefining: GWP and FDP Scores (The abbreviations represent the names listed in Table S9).

**Fig. S25**. Environmental scoring of process variables. Environmental scores for process variables in photorefining technology for xylan. (The abbreviations represent the names listed in Table S9).

**Table S13.** Final environmental score for the xylan processing technology.

| Impact category | Unit | Final environmental scores for the three processes. | | | | |  |
| --- | --- | --- | --- | --- | --- | --- | --- |
|  |  | fermentation | | pyrolysis | | photorefining | |
| Global warming (GWP) | kg CO_2_ eq | 31.3371 | 22.2739 | | 12.7200 | |  |
| Stratospheric ozone depletion (SOD) | kg CFC_11_ eq | 0.0001 | 0.0000 | | 0.0000 | |  |
| Ionizing radiation (IR) | kBq Co-60 eq | 0.4001 | 0.2986 | | 0.5520 | |  |
| Ozone formation, Human health (OFH) | kg NO_x_ eq | 0.0869 | 0.0628 | | 0.0321 | |  |
| Fine particulate matter formation (FPM) | kg PM_2.5_ eq | 0.0637 | 0.0470 | | 0.0270 | |  |
| Ozone formation, Terrestrial ecosystems (OFT) | kg NO_x_ eq | 0.0874 | 0.0631 | | 0.0326 | |  |
| Terrestrial acidification (TA) | kg SO_2_ eq | 0.1794 | 0.1303 | | 0.0664 | |  |
| Freshwater eutrophication (FE) | kg P eq | 0.0170 | 0.0128 | | 0.0070 | |  |
| Marine eutrophication (ME) | kg N eq | 0.0014 | 0.0009 | | 0.0024 | |  |
| Terrestrial ecotoxicity (TE) | kg 1,4-DCB | 52.8504 | 42.1161 | | 46.5106 | |  |
| Freshwater ecotoxicity (FEO) | kg 1,4-DCB | 0.8065 | 0.6234 | | 0.4779 | |  |
| Marine ecotoxicity (MEO) | kg 1,4-DCB | 1.1418 | 0.8827 | | 0.6804 | |  |
| Human carcinogenic toxicity (HCT) | kg 1,4-DCB | 1.5419 | 1.1578 | | 0.6370 | |  |
| Human non-carcinogenic toxicity (HNC) | kg 1,4-DCB | 24.5244 | 19.0986 | | 15.1289 | |  |
| Land use (LU) | m^2^a crop eq | 0.7137 | 0.4858 | | 0.4158 | |  |
| Mineral resource scarcity (MRS) | kg Cu eq | 0.0572 | 0.0370 | | 0.0464 | |  |
| Fossil resource scarcity (FDP) | kg oil eq | 7.5072 | 5.4696 | | 4.1751 | |  |
| Water consumption (WC) | m^3^ | 0.5466 | 0.4110 | | 0.4478 | |  |

**Fig. S26**. GWP and FDP for the final environmental scores of the three technologies (fermentation, pyrolysis and photorefining).

**Fig. S27**. Final environmental scores for three processes (fermentation, pyrolysis and photorefining).

**Fig. S28**. GWP score of process variables. Environmental scores for process variables in photorefining technology for xylan.

**Fig. S29**. FDP score of process variables. Environmental scores for process variables in photorefining technology for xylan.
